# Open chromatin profiling identifies AP1 as a transcriptional regulator in oesophageal adenocarcinoma

**DOI:** 10.1371/journal.pgen.1006879

**Published:** 2017-08-31

**Authors:** Edward Britton, Connor Rogerson, Shaveta Mehta, Yaoyong Li, Xiaodun Li, the OCCAMS consortium, Rebecca C. Fitzgerald, Yeng S. Ang, Andrew D. Sharrocks

**Affiliations:** 1 School of Biological Sciences, Faculty of Biology, Medicine and Health, University of Manchester, Manchester, United Kingdom; 2 School of Medical Sciences, Faculty of Biology, Medicine and Health, University of Manchester, Manchester, United Kingdom; 3 Christie Hospital, Manchester, United Kingdom; 4 MRC Cancer Unit, Hutchison/MRC Research Centre, University of Cambridge, Cambridge, United Kingdom; 5 GI Science Centre, Salford Royal NHS FT, University of Manchester, Stott Lane, Salford, United Kingdom; Duke-National University of Singapore Graduate Medical School, SINGAPORE

## Abstract

Oesophageal adenocarcinoma (OAC) is one of the ten most prevalent forms of cancer and is showing a rapid increase in incidence and yet exhibits poor survival rates. Compared to many other common cancers, the molecular changes that occur in this disease are relatively poorly understood. However, genes encoding chromatin remodeling enzymes are frequently mutated in OAC. This is consistent with the emerging concept that cancer cells exhibit reprogramming of their chromatin environment which leads to subsequent changes in their transcriptional profile. Here, we have used ATAC-seq to interrogate the chromatin changes that occur in OAC using both cell lines and patient-derived material. We demonstrate that there are substantial changes in the regulatory chromatin environment in the cancer cells and using this data we have uncovered an important role for ETS and AP1 transcription factors in driving the changes in gene expression found in OAC cells.

## Author summary

Oesophageal adenocarcinoma is one of the ten most prevalent forms of cancer and is showing a rapid increase in incidence and yet exhibits poor survival rates. Understanding the molecular causes of this type of cancer will enable us develop more effective treatment strategies which will improve survival rates. Here we have investigated how the genes in cancer cells are packaged into chromatin. We then compare this packaging to normal cells and use this information to identify the molecular causes leading to changes in chromatin packaging in cancer cells. We have identified a regulatory factor called AP1 that acts as a molecular switch to alter gene expression and hence cause cells to adopt a cancer fate. Importantly either this regulatory factor or a coregulatory protein from the ETS family is upregulated in the majority of cancer cells. Our study has therefore uncovered an important regulatory pathway that is commonly activated in oesophageal adenocarcinoma cells.

## Introduction

The incidence of oesophageal adenocarcinoma (OAC) in the Western world is increasing and five and ten year survival rates remain low [1,2]. In comparison to many other cancers, there is a general lack of knowledge about biomarkers and potential therapeutic targets, contributing to the poor prognosis for patients with this disease. More recently, this situation has improved with several studies employing genome-wide approaches to further our understanding of the molecular defects in OAC. Several microarray studies have identified gene signatures that are of prognostic value [3,4] and recent genome-sequencing studies have uncovered new mutations and genomic rearrangements commonly found in OAC samples and associated these with disease progression [5–9]. Importantly many of the defects detected are in genes encoding proteins that affect chromatin structure such as mutations found in genes encoding the chromatin remodeling complex components ARID1A and SMARCA4 [5,6]. However, these mutations are also detected in the pre-cancerous Barrett’s oesophagous stage, whereas transcription factor mutations or amplifications occur more often after the transition to adenocarcinomas [9]. These findings suggest a model for adenocarcinoma development that involves alterations to the chromatin structure accompanied with reprogramming of the gene expression profiles driven by changes in transcription factor activity.

Several studies have implicated different transcription factors as important drivers of oesophageal cancer, chiefly due to their overexpression in OAC cell lines and/or patient derived OAC samples. Well studied examples include GATA6 [10,11], and FOXM1 [12,13]. Members of the ETS transcription factor family are often implicated as oncogenic cancer drivers, and this is best exemplified by the role of ERG, and PEA3 subfamily members in prostate cancers [reviewed in 14]. Indeed, members of the PEA3 subfamily, ETV1, ETV4 and ETV5 have been implicated in a wide range of cancers [reviewed in 15] and ETV4 has been implicated in oesophageal adenocarcinomas [16]. However, the target genes and mechanisms used by PEA3 transcription factors to control their expression in OAC are not known. Indeed, despite these studies, our understanding of the transcriptional control networks that are deregulated in oesophageal cancer is not well developed.

In this study, we investigated the changes that occur in the regulatory chromatin landscape in oesophageal adenocarcinoma by an unbiased approach using ATAC-seq. We identified AP1 and ETS transcription factors as important regulators in OAC cells and targeted ChIP-seq analysis combined with knockdown experiments reinforced the role of the ETS protein ETV1 in driving OAC-specific gene expression programmes. Similarly, loss of function approaches validated a regulatory role for AP1. Our results therefore demonstrate an important role for AP1 in OAC and part of its action is through a regulatory module containing AP1 and PEA3 subfamily ETS transcription factors. Importantly one or both of these factors are commonly upregulated in patient-derived OAC samples, and both factors are implicated in regulating the active open chromatin environment in these cells.

## Results

### Identification of ETV1 binding sites in OAC cells

Our previous studies focussed on ETV4 and its role in OAC but also demonstrated that the closely related transcription factor ETV1 is upregulated in OAC [16]. We were unable to identify any antibodies that were suitable for ChIP-seq analysis of ETV4, therefore we instead focussed on ETV1. We verified that ETV1 is important for OAC cell growth as depletion of ETV1 reduced the growth rate of both OE33 and OE19 cell lines (S1A and S1B Fig). To determine how this transcription factor might impact on OAC and identify the regulatory networks it participates in we used ChIP-seq analysis to identify the direct targets of ETV1. We focussed on OE33 cells because ETV1 is expressed to the highest level in OE33 cells among the OAC cell lines we tested (S1C Fig).

**Fig 1:**
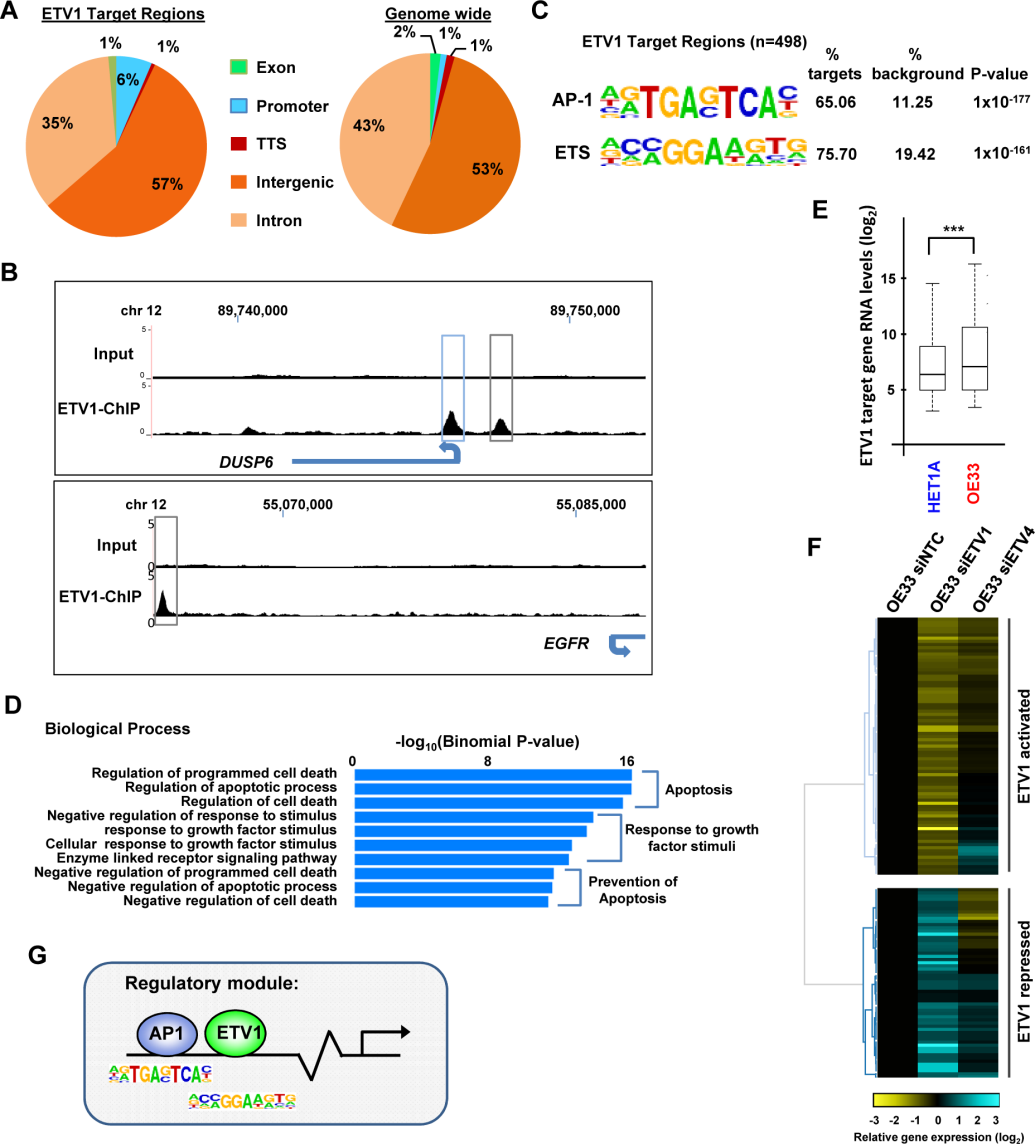
Identification of the ETV1 cistrome in oesophageal adenocarcinoma-derived OE33 cells. (A) Pie charts showing the genomic distribution of the ETV1 binding peaks (left) and the expected ratio of genomic regions (right) (promoter defined as +/- 1 kb from TSS). (B) UCSC browser track showing ETV1 binding peaks (bottom tracks) at the *DUSP6* and *EGFR* loci with enrichment over the input sample (top tracks). Intergenic (grey boxes) and promoter proximal (blue boxes) peaks are highlighted. (C) Top two motifs from *de novo* motif discovery at the ETV1 binding regions. (D) The top 10 gene ontology terms from the biological processes category of the genes associated with ETV1 binding regions. (E) Box plots showing the average gene expression levels from microarray analysis of the 398 direct ETV1 target genes in HET1A compared to OE33 cells. Horizontal line represents the median values; ***P-value = <0.001 (2 tailed T-test). (F) Heatmap showing gene expression changes of direct ETV1 target genes following siRNA-mediated ETV1 or ETV4 knockdown in OE33 cells. Only genes showing a fold change of >1.5 following depletion of ETV1 are shown. Data are normalised to OE33 treated with a non-targeting control siRNA (siNTC), and clustered. (G) Model showing a potential regulatory module acting at ETV1 binding elements consisting of AP1 and ETV1 transcription factors.

The *DUSP6* promoter was demonstrated to be target for PEA3 family proteins in two previous ChIP-seq studies in ECC-1 endometrial carcinoma cells [17] and PC3 prostate cancer cells [18](S2B and S2C Fig). We therefore used the *DUSP6* promoter as a positive control to optimise the ETV1 ChIP protocol (S2A and S2D Fig). Two ChIP-seq replicate experiments were performed in OE33 cells and the binding peaks showed a high degree of concordance (86% overlap at q value <0.01). We therefore merged the two datasets and recalled the peaks giving a total of 498 ETV1 binding regions (q-value <0.01)(S1 Table). Very few of these binding sites are located in proximal promoters (ie +/- 1kb from the TSS) and instead are largely located in intronic and intergenic regions, suggestive of enhancer binding (Fig 1A). ETV1 binding was identified in the *DUSP6* promoter region and other newly identified ETV1 targets such as an intergenic region associated with the *EGFR* locus (Fig 1B). One of the two highest scoring motifs found in the ETV1 binding regions was closely related to the CCGGAA core motif recognised by ETS transcription factors [19], and was found in 76% of targets, thereby validating the quality of the ChIP-seq data (Fig 1C). However, unexpectedly, the highest scoring over-represented motif contained the core sequence TGA^G^/_C_TCA which is the recognition site for AP1 transcription factors and this was found at a very high frequency in 65% of ETV1 binding regions (Fig 1C). Several other motifs were also enriched but at much lower levels (S3 Fig).

Next we associated the ETV1 peaks with the closest gene (398 genes in total; S1 Table) and looked for enriched gene ontology terms. All of the top enriched biological process terms are related to controlling apoptosis and the response to growth factor signalling (Fig 1D), consistent with the cellular growth defects we observed upon ETV1 depletion. We compared the expression of these genes between HET1A (non-cancerous, SV40 large T antigen transformed oesophageal cells) and OE33 (OAC-derived cells) by microarray analysis (S2 Table). The ETV1 target genes are generally expressed to higher levels in OE33 cells (Fig 1E), which is suggestive of a role for ETV1 in promoting cancer cell-specific gene expression. Consistent with this, when we depleted ETV1 in OE33 cells, microarray analysis identified 121/398 ETV1-associated target genes (30%) which showed significant differences in expression (>1.5 fold difference; p<0.05). Of these differentially regulated genes, the largest proportion 75/121 (62%) showed decreased expression following ETV1 knockdown (Fig 1F). We validated these results by RT-qPCR analysis of a panel of targets using a different ETV1 siRNA construct which was not present in the original siRNA pool. Importantly, the majority of the targets (7/9) showed similar downregulation in the presence of either the new siRNA or the original pool of siRNAs against ETV1 (S4 Fig). Many of the ETV1 regulated genes also showed consistent deregulation upon depletion of the related transcription factor ETV4, suggesting some level of functional overlap (Fig 1F).

Collectively, these data are therefore consistent with ETV1 having an important role as a direct transcriptional activator of dozens of genes in OAC-derived cells largely through distal regulatory elements. DNA sequence motif analysis is strongly suggestive that AP1 transcription factors likely function alongside ETV1 in this role (Fig 1G).

### ATAC-seq identifies AP1 factors as potential OAC-specific transcriptional regulators.

**Fig 2:**
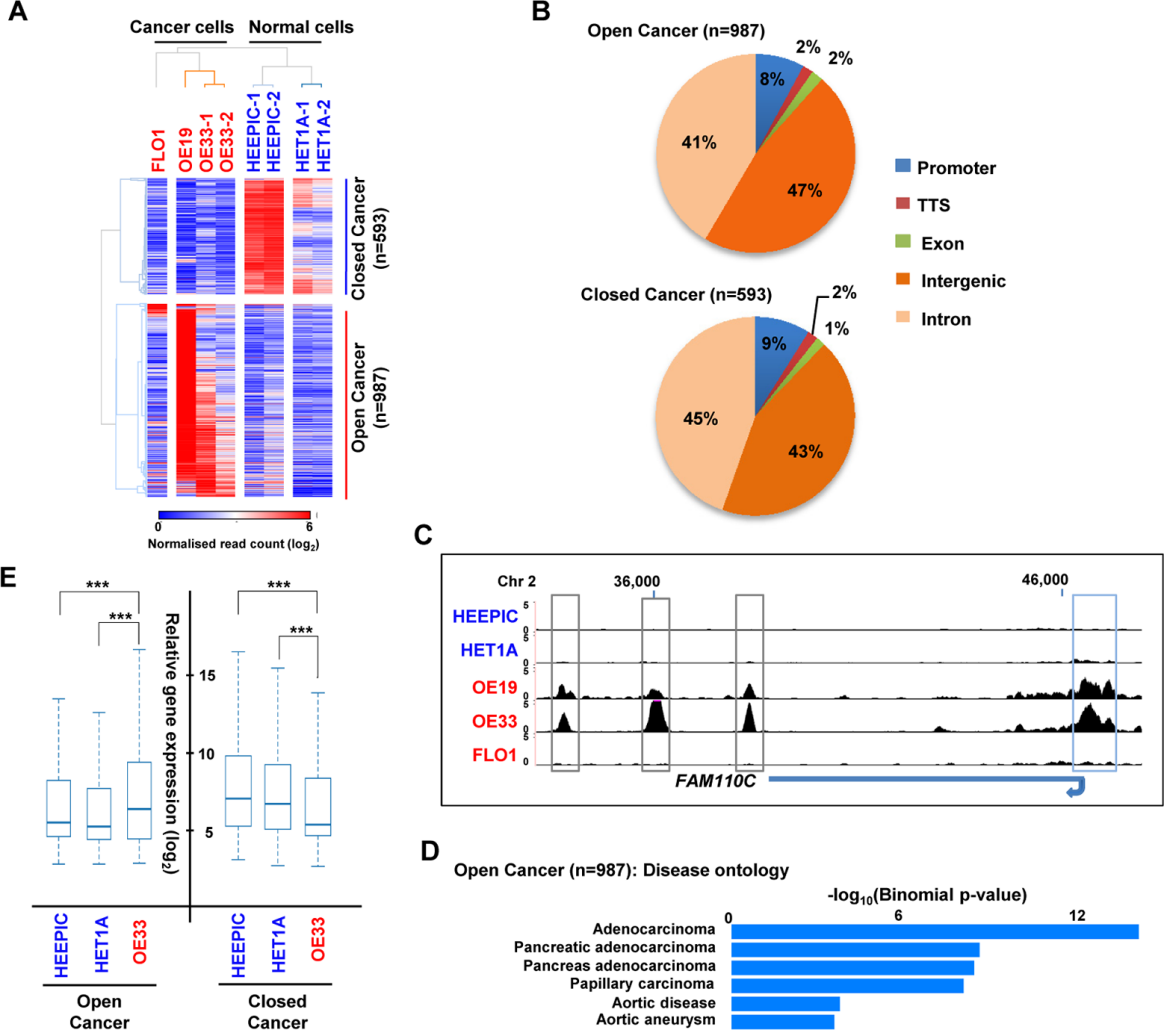
ATAC-seq reveals open chromatin regulatory regions in OAC cell lines. (A) Heatmap of normalised Tn5 cleavage events (log_2_) in 500 bp regions surrounding the summits of the significant differentially accessible regions (+/- linear fivefold change, p<0.05) between non-cancer and cancer cell lines. Replicate data are shown for three of the cell lines. Data are hierarchical clustered using 1-Pearson’s correlation and grouped according to either more “open” or “closed” in cancer. (B) Pie chart showing the genomic distribution of the differentially accessible regions (promoter = +/- 1 kb from TSS). (C) UCSC browser track showing open chromatin regions at the *FAM110C* locus in the indicated cell lines. Intergenic (grey boxes) and promoter proximal (blue boxes) peaks are highlighted. (D) The top 6 gene ontology terms from the disease category of the genes associated with the differentially accessible regions more open in cancer cells. (E) Box plots of expression (log_2_) from microarray data of the nearest gene to differentially accessible peaks in the indicated cell lines. Horizontal line indicates median. *** = P-value <0.001 (t test).

Many of the ETV1 binding regions are located in distal intergenic regions. To begin to establish whether these are associated with open and hence potentially “active” chromatin regions, we mapped the open chromatin regions in OE33 cells using ATAC-seq. We also mapped the open chromatin regions in two other OAC-derived cell lines, OE19 and FLO1 [20] and compared these to two non-cancer-derived “normal” oesophageal cell lines, HET1A and HEEPIC. Replicate experiments demonstrated that the experiments were highly reproducible (S5A–S5C Fig) and comparisons with histone marks indicated that the distal intergenic regions identified by ATAC-seq are associated with active enhancer regions (ie marked with H3K27ac and H3K4me1; S5D–S5F Fig). To identify regions which are more accessible in cancer cells and hence potentially more active, we first combined all of the reads from the three OAC-derived cell lines and the two normal cell lines and recalled the peaks of open chromatin which are characteristic of the cancer or normal cell lines. This gave us a comprehensive set of regions that are accessible in one or more cell lines. We then took the top 50,000 regions and used a 500 bp region around the summit for these peaks as an accessibility window across which we determined the variations in accessibility between cell types. We identified 1580 regions which are differentially accessible between the normal and the cancer phenotype (5 fold difference; p<0.05); 987 regions were more accessible in the cancer condition and 593 were more accessible in the normal condition (S3 Table). Clustering analysis using these differentially accessible peaks demonstrated that the replicate experiments cluster tightly and the normal and cancer-derived cell lines are also clustered together (Fig 2A). There are however differences apparent among the cancer samples with OE19 and OE33 cells showing more similarity to each other than to FLO1 cells which are clustered independently (Fig 2A, left).

Next we identified the locations of the differentially accessible peaks and found that the majority are located in intergenic and intronic regions (88%) with only 8–9% found in promoter regions (Fig 2B). Examples of the promoter-proximal and intergenic peaks clearly demonstrate the cell type specificity in either subsets of the OAC-derived cell types (Fig 2C) or normal oesophageal cells (S6A Fig). We associated the differentially accessible regions to the nearest gene and performed gene ontology analysis. The results are consistent with the expected effects of deregulating the putative target genes ie regions which became open and active in cancer cells were associated with genes belonging to GO terms encompassing various adenocarcinomas (Fig 2D) and expression profiles associated with various intestinal organs (S6B Fig). Conversely, the regions which were closed in cancer cells are associated with different GO terms, chiefly associated with various signaling processes (S6C Fig). Finally we asked whether the regions which change accessibility are indicative of changes in gene expression between normal and cancer cells. Importantly we demonstrate that regions which become more open in cancer cells are associated with genes which are more highly expressed in OAC-derived OE33 cells (Fig 2E). On the contrary, regions which become closed in cancer are associated with genes whose expression is lower in the OE33 cells (Fig 2E). The analysis of differentially accessible peaks identified by ATAC-seq therefore reports on regulatory events that likely contribute to the changes in gene expression observed in cancer cells.

**Fig 3:**
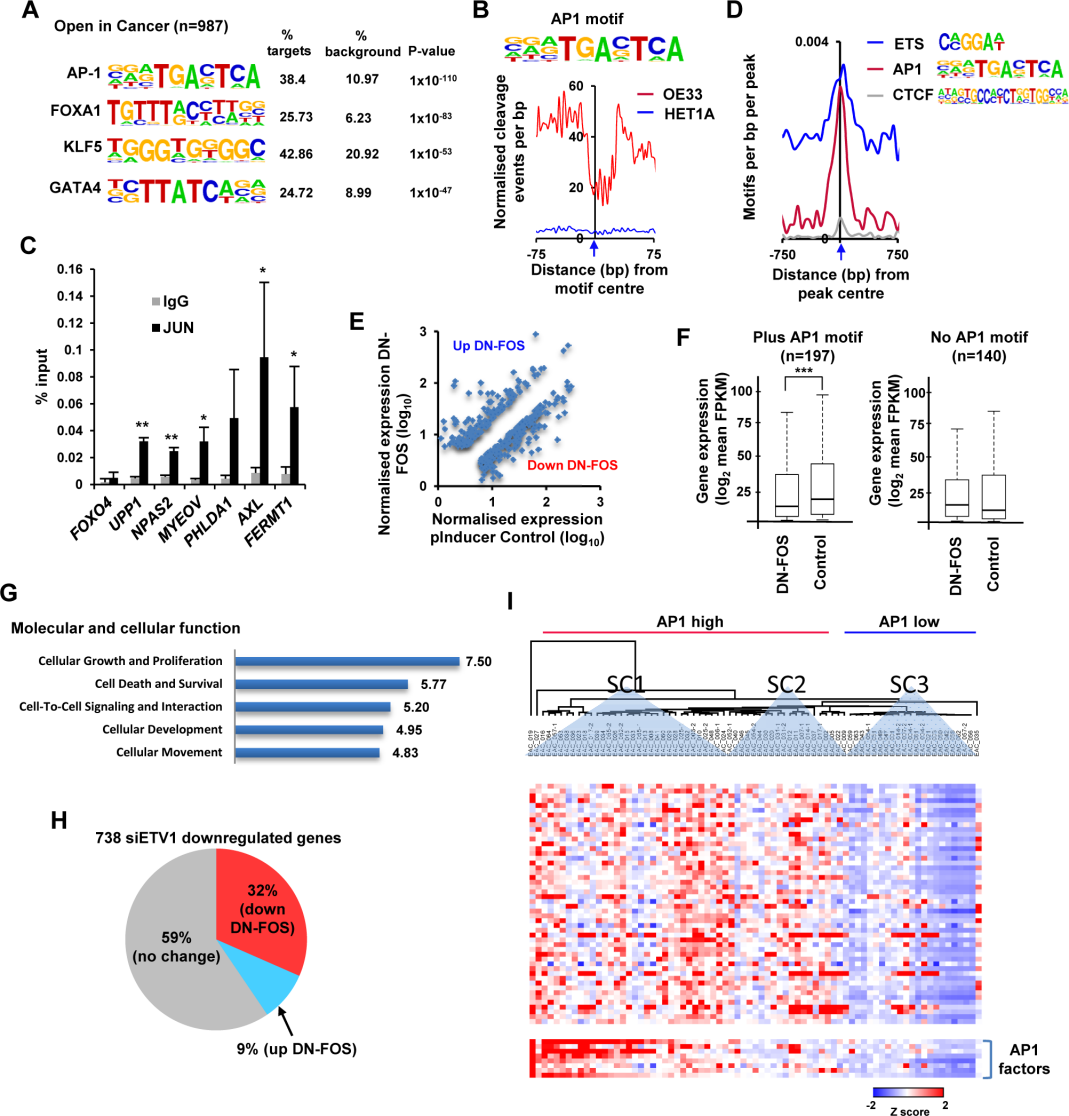
Identification of AP1 as a potential regulator of differential chromatin accessibility in OAC-derived cells. (A) Top four motifs from *de novo* motif discovery at the 987 differentially accessible chromatin regions (+/- 250 bp from the centre) which are more open in cancer. (B) Plot of normalised Tn5 cleavage events +/-75bp from the motif centre (blue arrow) around the AP-1 motifs found in differentially accessible chromatin regions that are more open in cancer cells. Data are plotted from OE33 (red) and HET1A (blue) cells. (C) ChIP-qPCR analysis of JUN binding to accessible chromatin regions associated with the indicated genes in OE33 cells. Non-specific IgG is used as a control and the *FOXO4* locus is a control region not bound by AP1. Data are shown as means ±SD (n = 3); * = P-value <0.05 and ** = P-value <0.01 (t test). (D) Relative density of the indicated motifs around the centre of the differentially accessible chromatin regions which are more open in cancer cells. Data are plotted as numbers of motifs per base pair across the regions. (E) Scatterplot showing genes which change significantly (linear 1.5 fold change, q<0.01, FPKM>10) and are either up- or down-regulated following DN-FOS expression. (F) Box plots of expression (log_2_) of nearest gene to differentially accessible peaks found to be more open in OE33 cells compared to HET1A cell lines. Data are partitioned according to whether the associated differentially accessible peak contains an AP1 motif, and are calculated using genes showing significant changes in expression (>1.5 fold; q-value <0.01) in OE33 cells transduced with viral vectors encoding DN-FOS or empty vector (control). Horizontal line indicates median; *** = P-value <0.001 (t test). (G) The top 5 gene ontology terms from the molecular and cellular function category of the genes showing significantly changed expression in cells transduced with lentiviruses encoding DN-FOS. (H) Pie chart showing the effect of DN-FOS transduction on the expression of the 738 genes which are downregulated by treatment with siRNA against ETV1 (2 fold change). Up and downregulated genes are defined as >1.5 fold change (q-value <0.01). (I) Expression of putative AP1 target genes across OAC-derived tissue samples (microarray data, GSE13898; [4]). AP1 target genes are selected based on their association with nearby regions that are both more open in cancer cells and also contain an AP1 motif and in addition, exhibit reduced expression following DN-FOS expression (>1.3 fold). Data are row Z normalised and subjected to Eucladian clustering and three main subclusters are highlighted (SC1-3). SC3 delineates samples that show low level AP1 expression and low level target gene expression.

To identify potential regulatory factors responsible for generating the open chromatin regions in OAC-derived cells, we searched for sequence motifs that were enriched in the peaks that were either more open or more closed in cancer cells. The top ranked motif in the regions becoming more accessible in cancer cells was the AP1 transcription factor recognition site TGA^G^/_C_TCA (Fig 3A). Several other over-represented motifs recognised by the transcription factors, FOXA1, KLF5 and GATA4 were also identified. Importantly, we could detect a binding footprint across the AP1 binding motifs specifically in the cancer-derived ATAC-seq data from OE33 cells, indicating that the motifs are likely occupied in a large number of regions (Fig 3B; S7B Fig). Somewhat counter-intuitively, AP1 motifs were also the highest ranked over-represented motifs in the regions showing lower accessibility in OE33 cancer cells (S7A Fig). However a much less pronounced footprint at these AP1 motifs was obvious in the non-cancerous HET1A cells (S6B Fig) suggesting lower occupancy levels. To validate AP1 occupancy we tested the binding of the AP1 subunit JUN to six regions which show enhanced accessibility in OAC cells and contain an AP1 motif. All of these regions exhibited JUN binding (Fig 3C). ETS motifs were not among the top ranked over-represented motifs in the chromatin regions that are more open in cancer cells. However, we searched for the degenerate versions of the ETS motif CC/AGGAA/T and found this motif to be highly enriched around the centre of these open chromatin regions, although the local background levels were higher than observed for AP1 (Fig 3D).

These data are indicative of a role for AP1 in acting through the open chromatin regions to drive increases in gene expression in cancer cells. To determine whether this was the case, we disrupted AP1 activity by transducing OE33 cells with a lentivirus expressing a dominant-negative version of FOS (DN-FOS) which forms non-DNA binding heterodimers with endogenous JUN family members [21]. We observed 1527 upregulated and 2087 genes downregulated following DN-FOS expression (linear 1.5 fold change; q<0.01; FPKM >10)(Fig 3E; S4 Table). Importantly, of the 626 genes associated with the regions showing increased accessibility in OE33 cancer cells compared to HET1A cells and containing an AP1 motif, 27% show reduced expression in OE33 cells in the presence of DN-FOS. Next we partitioned the genes associated with regions that are more accessible in OE33 cancer cells into two groups according to the presence or absence of an AP1 motif. We found that there is a clear reduction in gene expression after expression of DN-FOS only in the group containing an AP1 motif (Fig 3F). These data are fully consistent with a model whereby AP1 is occupying and activating a significant proportion of the genes with regulatory regions which are becoming more accessible in cancer cells. Importantly inhibiting AP1 function affects cohorts of genes involved in cell growth, survival and movement which are all functions previously attributed to AP1 in other cellular environments ([22]; Fig 3G).

Given our identification of a regulatory module containing AP1 and ETS motifs, we asked whether ETV1 regulated genes were also regulated by AP1. Inhibition of AP1 function by DN-FOS inhibited a large proportion of the ETV1 activated genes (ie genes showing downregulation upon siETV1 treatment), with 32% (233/738) also showing downregulation after AP1 inhibition (Fig 3H; hypergeometric test, P = 3.8x10^-50^). The active chromatin state is usually coincident with the appearance of histone H3K27 acetylation. We therefore tested whether inhibition of AP1 activity by DN-FOS also affected the levels of this chromatin mark. At all four loci tested, DN-FOS reduced the levels of gene expression but with the exception of *MMP1*, failed to change the levels of H3K27ac (S8 Fig). Thus, the ETV1-AP1 binding module appears functionally relevant in OAC cells but is not generally required for maintaining histone acetylation levels.

Finally we investigated whether the genes controlled by AP1 in OE33 cells are relevant in the context of human OAC samples. To do this we defined a high confidence set of putative AP1 target genes, by first associating the differentially accessible peaks that are found to be more accessible when comparing all tumour and normal cell lines genes with the nearest gene and then selecting those peaks containing an AP1 motif within a 500 bp window of the peak summit. We then filtered for a reasonable level of expression (>5 FPKM), and >1.3 fold reduction in expression following DN-FOS expression which gave us a total of 58 genes which are high confidence direct AP1 targets. We then examined the expression of these genes in a panel of OAC samples [4] and plotted their expression according to AP1 status in each sample. The resulting heatmap demonstrated a clear correlation with AP1 expression levels across many of the target genes, with higher levels of AP1 expression generally being associated with high level target gene expression (both generally red on the left side of the heatmap) and reciprocally, low AP1 levels are associated with low target gene expression (both generally blue on the right side of the heatmap) (Fig 3I; S9 Fig).

Collectively, this data identifies the AP1 transcription factor as an important player in driving the gene expression profiles found in OAC cells.

### ETV1 is associated with open chromatin in OAC cells

**Fig 4:**
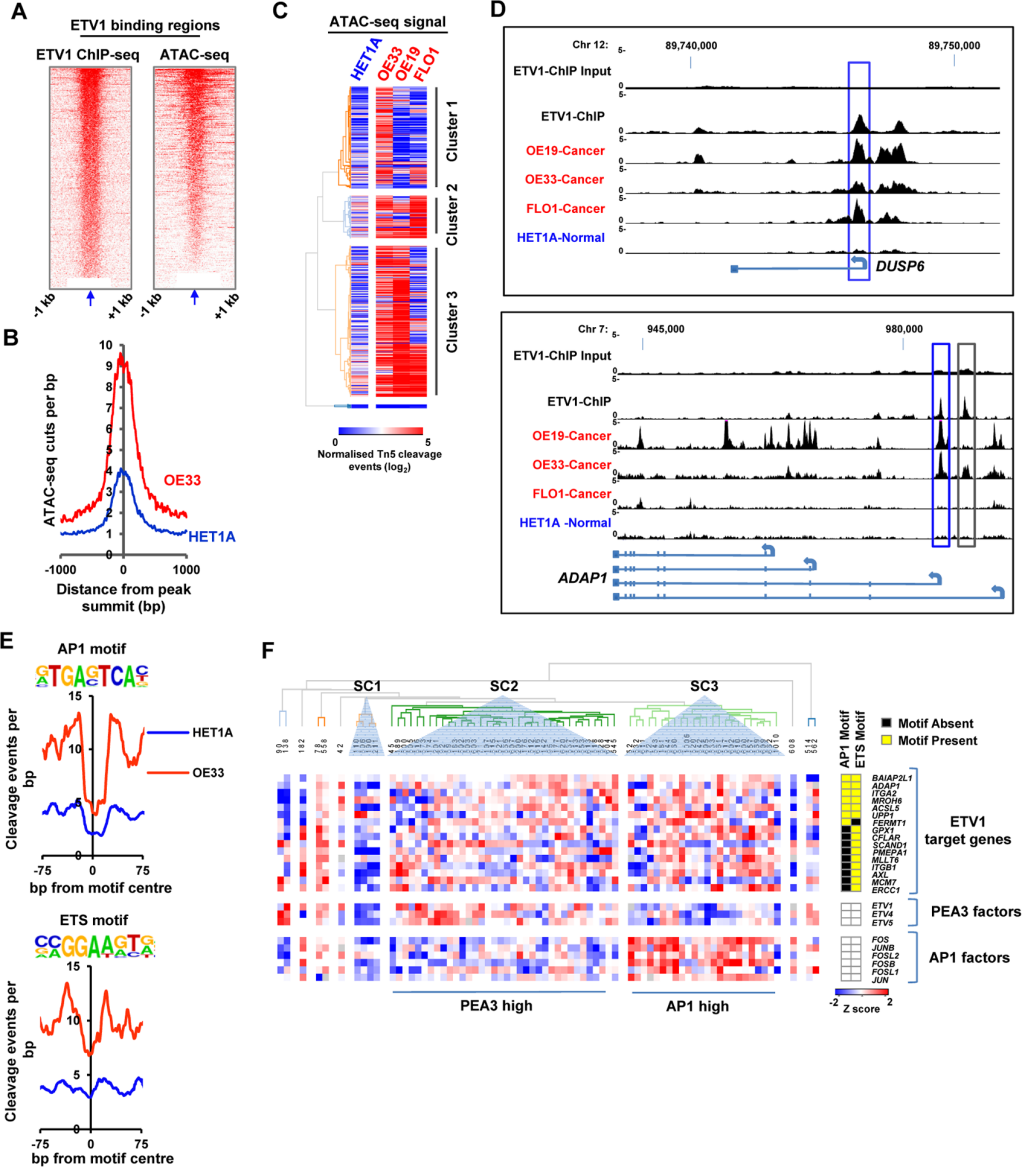
ETV1 binding regions are associated with open chromatin. (A) ChIP-seq (left) and ATAC-seq (right) tag densities compared in a 2 kb window around summit (indicated by arrow) of ETV1 binding regions. (B) Plot of normalised Tn5 cleavage events +/-1 kb from the peak summits of the ETV1 binding regions. Data are plotted from OE33 (red) and HET1A (blue) cells. (C) Heat map showing the relative accessibility (normalised ATAC-seq reads) found in 500 bp windows surrounding the summit of ETV1 binding regions in the HET1A (normal derived) cell line and the three cancer derived cell lines OE33, OE19 and MFD-1. Replicates were merged for OE33 and HET1A. Regions are clustered using hierarchical clustering. (D) UCSC browser track showing ETV1 binding peaks compared to input sample in OE33 cells (top two tracks) at the *DUSP6* and *ADAP1* loci and ATAC-seq signal in the same regions in the OE19, OE33, FLO1 and HET1A cells. Intragenic (grey boxes) and promoter proximal (blue boxes) peaks are highlighted. (E) Plot of normalised Tn5 cleavage events +/-75bp from the motif centre around the AP-1 (left) and ETS (right) motifs found at ETV1 binding regions. Data are plotted from OE33 (red) and HET1A (blue) cells. (F) Heatmap of relative gene expression data for the indicated ETV1 target genes and PEA3 and AP1 family transcription factors from 73 OAC biopsy samples. Target genes were selected based on being associated with an ETV1 binding region which is open in OE33 cancer cells and contains either an AP1 or ETS motif (or both) (indicated by yellow boxes). Data were generated by RT-qPCR on the BioMark HD System (Fluidigm) and are plotted as row Z scores of–ΔCT (*GAPDH* normalised) values. Data are clustered according to Pearson’s correlations and prominent subclusters indicated (SC1-3).

Having established the open chromatin architecture of OAC-derived cells, we next asked whether the ETV1 binding regions we identified through ChIP-seq analysis are associated with open and potentially active chromatin. There is a strong overlap between the level of ETV1 binding and the presence of open chromatin in OE33 cells (Fig 4A). Furthermore, the ETV1-bound regions show increased accessibility compared to the non-tumourigenic HET1A cells (Fig 4B), suggesting an association with the acquisition of open chromatin. Indeed, further comparisons between the accessibility of the ETV1 binding regions in HET1A cells versus three different OAC-derived cell lines identified three broad clusters of sites (Fig 4C; S5 Table). Almost all the ETV1 binding regions show greater accessibility in OE33 cells than in the HET1A cell line. Indeed, one cluster shows increased accessibility which was largely limited to the OE33 cell line. However, the second cluster shows generally increased accessibility in all of the OAC cell models and this pattern is exemplified by binding at the *DUSP6* promoter (Fig 4D, top) whereas the largest third cluster is defined by increased accessibility in both the OE19 and OE33 cell lines as observed at the *ADAP1* locus (Fig 4D, bottom). Several of the genes showing increased chromatin accessibility across all samples have previously been shown to be associated with cancer. These include *CTSB* in OAC [23], *BAIAP2L1* in ovarian cancer [24] and *BCAR1* in a range of cancers [25]. We also examined whether we could see any transcription factor footprints in the regions occupied by ETV1 and as expected we could see a clear footprint at the ETS binding motif that is recognised by ETV1 (Fig 4E). We also asked whether we could observe a footprint at the AP1 motif which was identified as the most significantly over-represented motif in the ETV1 binding regions. Again we were able to identify a clear footprint in OE33 cells and at both the AP1 and ETS motifs, the footprints were clearly more enhanced in OE33 cells compared to the HET1A cell line (Fig 4E). Thus in addition to motif presence, this data indicates that these motifs are occupied in cancer cells, and further supports the existence of combinatorial actions of ETV1 and AP1 in controlling gene expression in OAC-derived cells.

We next examined whether the presence of associated ETV1 binding and potential AP1 co-occupancy had any relevance to gene expression in the context of human tumour biopsy samples. A high confidence set of ETV1 target genes were selected based on being associated with an ETV1 binding region which is open in OE33 cancer cells and also contains either an AP1 or ETS motif (or both). Target gene expression was then examined and samples clustered according to similarity in expression patterns (Fig 4F). This identified three major clusters, one small one with generally low level gene expression and two with generally elevated expression levels. Interestingly the latter two clusters, SC2 and SC3, are characterised by high level expression of PEA3 or AP1 family transcription factors respectively, suggesting that overexpression of either one of these classes of transcription factors is sufficient for enhanced target gene expression.

Together these results demonstrate that ETV1 binding occurs concomitantly with AP1 and is generally associated with chromatin which becomes more open and active in cancer cells. However, while some regions are specific to OE33 cells, others are commonly activated in several OAC-derived cell lines.

### AP1 and PEA3 family transcription factors are overexpressed in patient-derived OAC samples

**Fig 5:**
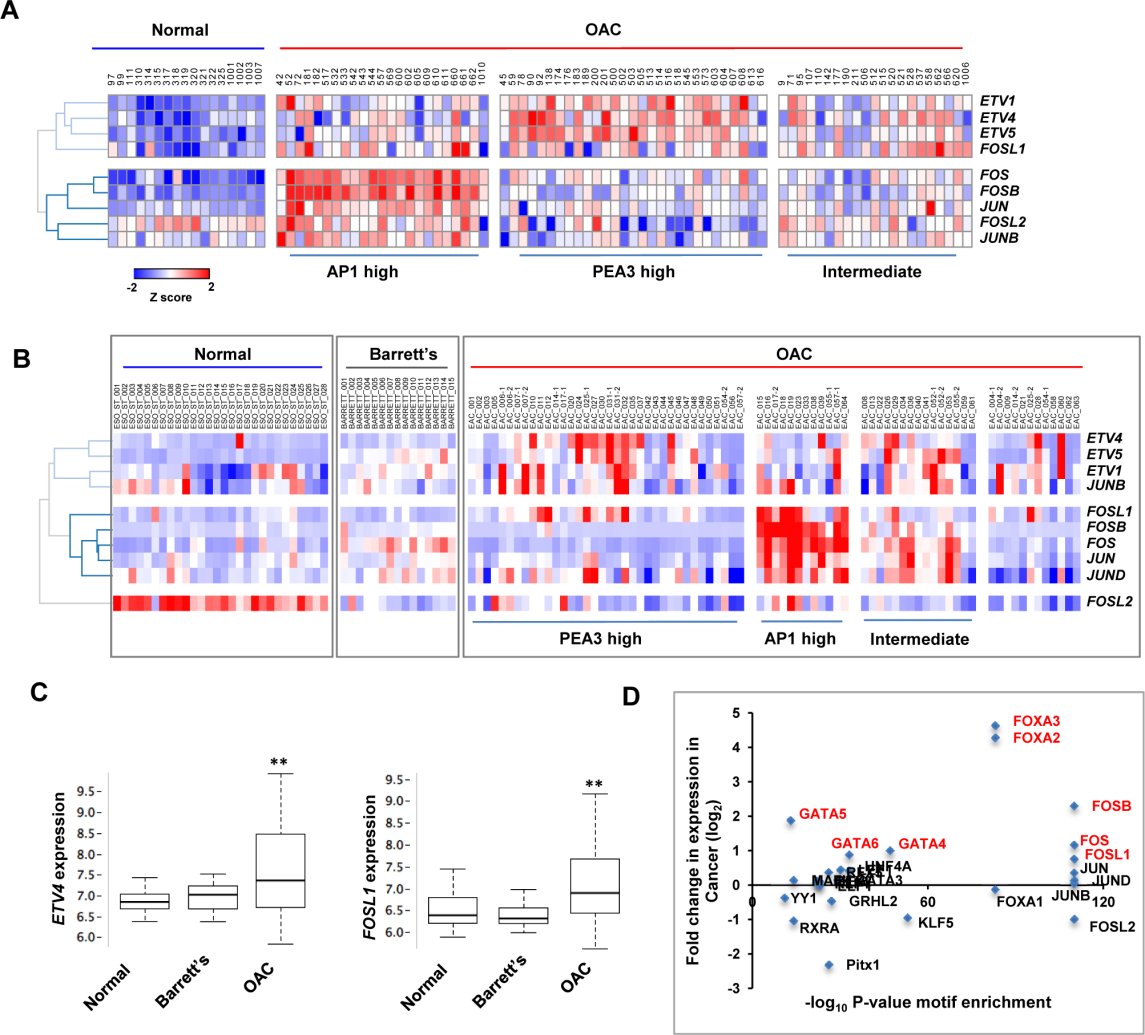
AP1 and ETS transcription factors are upregulated in human OAC samples. (A) Heatmap of relative gene expression data for the indicated PEA3 and AP1 family transcription factors from 73 OAC tumour and 17 normal oesophageal biopsy samples. Data were generated by RT-qPCR on the BioMark HD System (Fluidigm) and are plotted as row Z scores of–ΔCT (*GAPDH* normalised) values. (B) Heatmap of relative expression of the indicated PEA3 and AP1 family transcription factors from microarray gene expression data from normal, Barrett’s and OAC-derived tissue samples (GSE 13898; [4]). In both heatmaps, the rows are clustered by hierarchical clustering and the tumour samples by k-means clustering. (C) Box plots of expression (log_2_) of *ETV4* and *FOSL1* expression from microarray data [4] from normal, Barrett’s or OAC-derived samples. Horizontal line indicates median; ** = P-value <0.05 (t test). (D) To determine putative regulatory TFs, the enrichment of transcription factor motifs (-log_10_ binomial P-value)(x-axis) is plotted against fold difference (log_2_) in the mean gene expression (from microarray data; [4]) of the transcription factors which potentially bind to the motif, in cancer cells compared to normal cells (y-axis). Those highlighted in red show are deemed significant (linear 1.5 fold change in expression in the same direction as change in accessibility of the associated motif).

Our data from ChIP-seq and ATAC-seq studies in OAC-derived cell lines implicate ETV1 and AP1 transcription factors as important players in driving OAC-specific gene expression programmes. One prediction of these findings is that we should see greater expression of these transcription factors in patient-derived OAC samples compared to normal oesophageal tissue. AP1 is a heterodimeric transcription factor consisting of either homodimers of JUN family members (JUN, JUNB and JUND) or heterodimers of JUN family members with a FOS family member (FOS, FOSB, FOSL1 and FOSL2)[reviewed in 22]. ETV1 is structurally highly related to two other ETS proteins ETV4 and ETV5 which collectively form the PEA3 family and likely perform overlapping functions [15]. We therefore examined the expression of the individual AP1 family subunits and PEA3 family members in biopsies from 73 patients (S6 Table). In general all of the PEA3 and AP1 family members are expressed at low levels in normal samples with the exception of *FOSL2* (Fig 5A). The expression across OAC samples is varied and three broad clusters could be identified (Fig 5A). The first cluster has generally high levels of AP1 subunits whereas the second cluster has high level expression of PEA3 family subunits (including *ETV1*). Together these clusters represented the majority of the samples but 29% formed a third cluster which is characterised by high level expression of individual ETS and AP1 family subunits rather than coordinated upregulation of several family members. Overall patients with OAC therefore exhibited either coordinated upregulation of AP1 subunits or PEA3 subunits or combinations of individual subunits from the two transcription factor families.

To verify these findings, we also interrogated a published microarray study on OAC-derived samples which also included data from the pre-cancerous Barrett’s oesophagus stage [4]. Again, we observed generally low level expression of both PEA3 and AP1 family members in normal tissue with the exception of *FOSL2* which is higher (Fig 5B). Low level expression of all of the genes encoding these transcription factors was generally observed in samples from patients with Barrett’s oesophagus. In contrast, there are groups of patients with OAC which had either high level expression of one or more genes encoding PEA3 family members or subunits of the AP1 complex, and others that had mixed upregulation of both types of transcription factors. Interestingly when looking at average expression of individual transcription factors across all samples, the patterns we observe by comparing the entire families are less obvious although *ETV4* and *FOSL1* are generally overexpressed in the context of OAC (Fig 5C; S10 Fig). Collectively, these results are broadly in keeping with what we observed from our own patient cohort and indicate that upregulation of PEA3 family members or AP1 subunit components is characteristic of a large number of cancers from OAC patients.

Finally we wanted to understand the contribution of individual transcription factors to driving the activity of the open chromatin regions in OAC cells. We therefore correlated the mean gene expression changes for individual transcription factors in patient-derived OAC samples with the probability of observing their recognition motif in the regions showing increased accessibility in cancer-derived cell lines. This approach would enable us to determine whether any particular PEA3 family members or AP1 subunit components might be more important in this context. This analysis identified the FOS sub-family members, FOSL1, FOS and more markedly FOSB as the likely drivers of enhanced AP1 activity in patient samples (Fig 5D). We also identified FOXA2 and FOXA3 as potential regulators of OAC-specific gene expression. Conversely, FOSL2 was identified as a possible contributor to the changes in activity resulting in more open chromatin in normal cells (S11 Fig).

Overall, the expression data from patient-derived tumour material support a role for AP1 and PEA3 family members in contributing to OAC-specific gene regulatory events and in particular are strongly indicative of a role for members of the FOS-subfamily in this process. However, none of the clusters identified using AP1 and PEA3 family gene expression profiles associated with any particular patient clinicopathological characteristics, suggesting that overexpression of one or more of these factors is a general property of OAC.

### ATAC-seq in patient-derived samples confirms AP1 and ETS factors as potential OAC-specific transcriptional regulators

**Fig 6:**
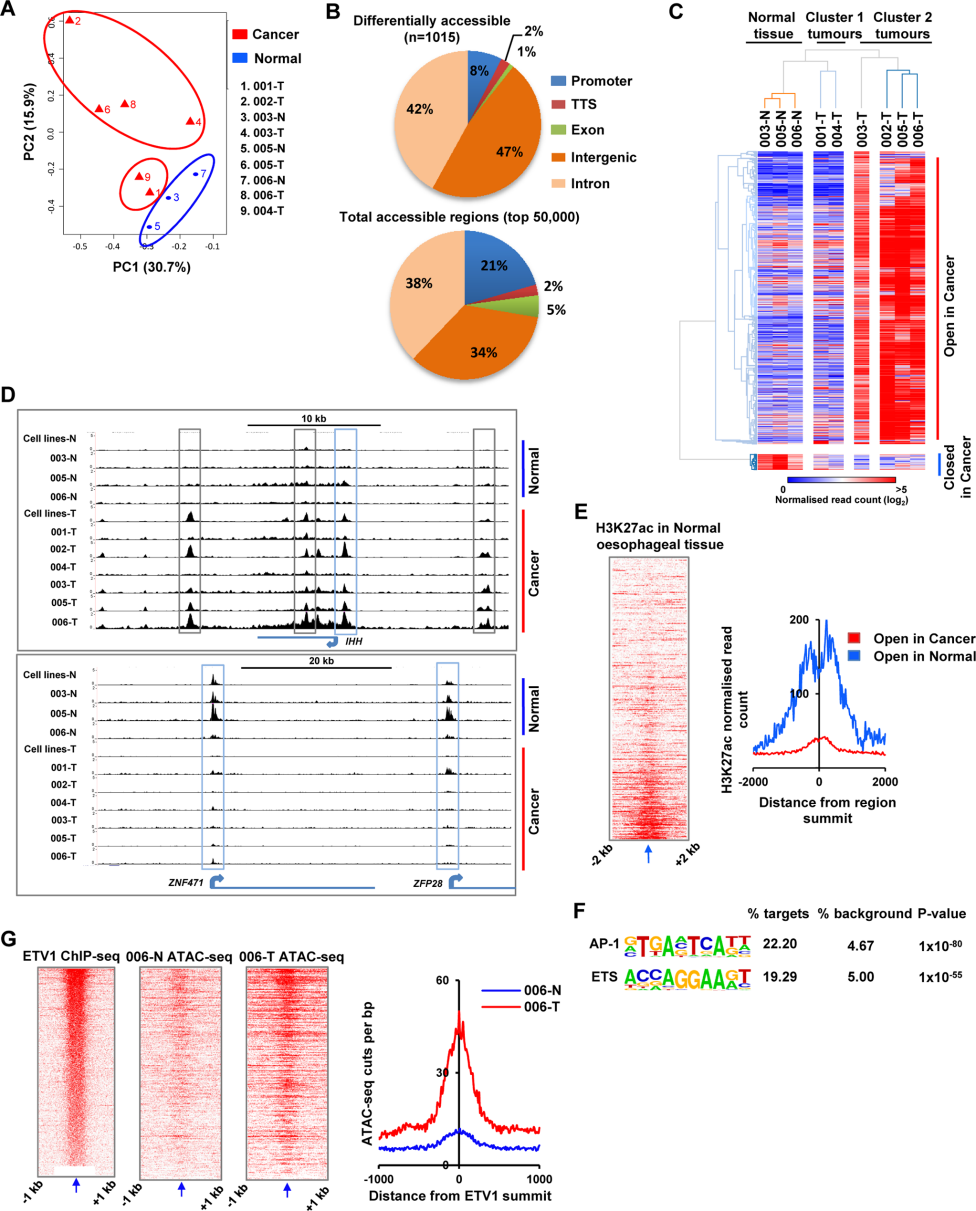
ATAC-seq reveals in patient samples identifies AP1 and ETS proteins as regulators of OAC. (A) PCA analysis of the 9 tissue samples across the top 50,000 accessibility regions derived from merging reads from all samples and recalling peaks using MACS2. Tumour samples are shown in red and normal samples in blue. (B) Pie chart showing the genomic distribution of the top 50,000 accessible regions across all tissue samples (bottom) or the 1015 differentially more accessible regions (top) in OAC cancer samples (promoter = +/- 1 kb from TSS). (C) Heatmap of normalised Tn5 cleavage events (log_2_) in 500 bp windows at the regions showing significant differential accessibility between normal and tumour tissue samples (+/- linear fivefold change, P<0.05). Data are subjected to hierarchical clustering using 1-Pearson’s correlation. (D) UCSC browser track showing open chromatin regions at the *IHH*, *ZNF471* and *ZFP28* loci in the indicated patient-derived normal and tumour tissue samples. Intergenic and intragenic (grey boxes) and promoter proximal (blue boxes) peaks are highlighted. Aggregated data from either two normal (-N) or three OAC (-T) cell lines is also shown. (E) Heatmap (left) and average tag profile (right) of normalised H3K27ac ChIP-seq tag density (GSM1013127) in normal oesophageal tissue (shown in a +/- 2 kb region around summit of each differentially accessible peak). Average profiles are shown for regions that are either more open in cancer (red line) or more open in normal (blue line) samples. (F) AP-1 and ETS motifs identified via de novo motif discovery, at regions that are more open in cancer (n = 962) against CpG matched background. (G) Heatmaps (left) and average ATAC-seq cleavage events (right) of ATAC-seq tag density in normal (blue line) and tumour (red line) samples from patient 006. Data are shown in a +/- 1 kb region relative to the summit of the ETV1 binding peaks defined by ChIP-seq in OE33 cells. Regions in the heatmaps are ranked according to ETV1 ChIP-seq signal (shown on the left).

Our data using cancer-derived cell lines, combined with gene expression data from cancer patients provides a compelling model which points to AP1 and PEA3 family members in driving OAC-specific gene expression. To further support this model, we used ATAC-seq to interrogate the regulatory open chromatin landscape of OAC-derived samples (3 normal samples and 6 OAC samples) and searched for evidence for regulation by these transcription factors. First we merged the reads from all the tissue samples both normal and cancer. We then identified the top 50,000 peaks from these merged reads and performed principal component analysis (PCA). The normal samples (blue) cluster together whereas the tumour samples (red) separated from the normal samples (Fig 6A). Furthermore on PC2 the tumours appear to separate into two groups with ATAC-001T and ATAC-004T being closer to the normal tissue samples and the others representing a distinct group which show similarity predominantly on PC2. To extend these results we identified the regions which are differentially accessible in cancer cells, by combining all of the reads from either the six OAC-derived samples or the three normal samples and recalled the peaks of open chromatin which are characteristic of the cancer or normal samples. This identified 1015 regions that showed significant differential accessibility (5 fold difference in tag counts; p-value <0.05) between the normal and the cancer samples. 964 regions are more open in the cancer cells and 52 more open in the normal cells indicating that the cancer cell genome is generally in a more open state (S7 Table). The genomic distribution of these regions is virtually identical to that observed in cell line models with the majority in putative intronic and intergenic regulatory regions with the biggest enrichments in the intergenic regions (Fig 6B). Of the 956 regions which exhibit increased accessibility in tumour tissue, 130 (14%) are also identified in the context of enhanced opening commonly found in OAC-derived cell lines. We then generated a heatmap of these regions in individual samples and probed their relationships using hierarchical clustering (Fig 6C). The normal samples all clustered together and the tumour samples were split into two groups with four tumours (cluster 2; ATAC-002T, ATAC-003T, ATAC-005T and ATAC-006T) appearing to have a similar pattern of increased accessibility across the samples whereas the other two tumour samples ATAC-001T and ATAC-004T (Cluster 1) cluster separately and appear to be closer to the normal tissue samples. Thus both clustering and PCA indicates the presence of two cancer subtypes. There are no obvious clinicopathological variables that segregate with the two clusters, but due to the small sample size more samples are needed to make any definitive conclusions.

The differentially accessible regions also point to potential differences in the underlying biology of OAC, as after associating these regions with the nearest gene, GO analysis identified various cancers and abnormalities in intestinal morphology as the most enriched categories (S12 Fig). By focusing in on the associated genes we are also able to pinpoint the location of potential regulatory elements such as the promoter proximal, intronic and distal elements associated in cancer cells with the *IHH* locus (Fig 6D, top). The activation of the *IHH* locus is consistent with recent discovery of *IHH* overexpression in OAC [26]. Reciprocally we can observe promoter elements being extinguished in cancer cells as exemplified for *ZNF471* and *ZFP28* (Fig 6D, bottom). Importantly, the appearance and loss of these open regions corresponds to changes in gene expression of the associated loci. *IHH* expression is upregulated in the cancer cells from cluster 2 tumours where regulatory chromatin accessibility increases (S13A Fig). However *ZNF471* behaves in the opposite manner and is expressed at lower levels in cancer cells compared to matched normal tissue (S13B Fig). To provide evidence that the differentially accessible regions we identified are representative of active chromatin regions, we took advantage of a published ChIP-seq dataset for H3K27ac (an active chromatin mark) in normal oesophageal tissue. We then analysed the tag counts for this mark in the regions which are more open in either normal or cancer cells. As predicted, the regions which are more accessible in normal cells and hence are likely more active, exhibit substantially higher H3K27ac signal than those that are newly accessible in cancer cells (Fig 6E). We then asked whether the motifs recognized by AP-1 and PEA3 family members (ETS motifs) are over-represented in the regions becoming more accessible in cancer cells and found that both AP-1 and ETS motifs are highly enriched in these regions (Fig 6F). Importantly, footprinting analysis in the differentially open chromatin regions demonstrates potential occupancy of these motifs in cluster 2 OAC samples by both AP1 (S14A Fig) and ETS family members (S14B Fig), but provides little evidence for binding at these motifs in the normal cells. Finally, given the prevalence of ETS motifs we asked whether there was evidence for enhanced levels of open chromatin in cancer samples at regions occupied by ETV1 in the OE33 OAC cell line. We focused on the matched samples and found that elevated levels of open chromatin could be found around the ETV1 binding regions in two of the cancer samples; 006-T (Fig 6G) and 005-T (S15A Fig), with a much reduced effect in 003T where the expression of ETV1 is much lower (Fig 6G; S15A and S15B Fig).

Together these data therefore provide independent verification of our model that AP1 and PEA3 family members act combinatorically to control the regulatory chromatin network underlying the phenotypic gene expression patterns found in OAC cells.

## Discussion

Phenotypic changes in cancer cells are ultimately elicited by changes in their gene expression profiles. Alterations to the underlying regulatory chromatin landscape of cancer cells facilitate these changes [reviewed in 27]. Here, we have uncovered the regulatory open chromatin landscape of OAC cells, and used this to infer the transcriptional regulators that are responsible for driving OAC-specific gene expression programmes. We integrated this data with ChIP-seq analysis of the ETS Transcription factor ETV1. ATAC-seq analysis in OAC derived cell lines and samples from patients with OAC identified AP1 and ETS factors as potential regulators. Reciprocally, ETV1 ChIP-seq implicated AP1 as a potential regulatory factor in OAC. Our results therefore point to two different transcription factor families in directing these programmes; AP1 and members of the PEA3 subfamily of ETS proteins. This is suggestive of a model where AP1 plays a leading role in controlling OAC-specific gene expression and part of this activity is mediated through working alongside PEA3 subfamily members to control gene activation (Fig 1G). However, although the model suggests a close molecular association between these transcription factors, their binding sites show no obvious distance constraints that would imply a distinct binding mode that would drive cooperative DNA binding. Further studies are needed to establish whether there is a binding hierarchy between these factors. Loss of function experiments confirms the regulatory activities of both ETV1 and AP1 in this regulatory module. Importantly, consistent with this regulatory role, either AP1 subunits, PEA3 subfamily members or both are over-expressed in the majority of OACs. Although we identified these transcription factors through their association with cancer-specific open chromatin regions, it is currently unclear whether one of both of these transcription factors is responsible for driving chromatin opening. Our results indicate that AP1 is not required for maintaining “active” histone acetylation levels but further studies are required to determine the roles of these factors in establishing and long term maintenance of the regulatory chromatin landscape.

Previous studies have inferred regulatory interactions between AP1 and ETS transcription factors and extrapolated this in the context of different cancers. For example, studies on the viral enhancers and the stromelysin and collagenase promoters initially identified functional cooperativity between these transcription factor families [28–30]. Since then, genome-wide studies have inferred potential regulatory cooperativity between AP1 and other ETS factors such as ELK1 [31] and ERG [18] and in the latter case this association is thought to be important for the context of prostate cancer. Regulatory interactions between the PEA3 subfamily member ETV5 and the AP1 component JUN have been identified [32] and in the case of ETV1 and ETV4, the interactions with AP1 have been expanded into a genome-wide view of the global consequences of these interactions [18]. It is thought that overexpression of ETS and AP1 factors can mimic upstream RAS/ERK signalling events and drive tumourigenesis in this context. It is therefore interesting to note that we previously showed co-upregulation of ERK signalling and ETV4 expression in OAC [16] and that genes encoding members of the RAS/ERK signalling cascade such as EGFR and KRAS are often amplified in the transition from Barrett’s oesophagus to adenocarcinoma [9]. Here we identify a novel association between AP1 and ETS factors in oesophageal cancer, and in particular with PEA3 subfamily members. In the case of AP1, we generally see overexpression of many different subunits in OAC with the exception of FOSL2 whose expression decreases. This observation is intriguing given the fact that we also see a set of AP1-bound regions as potentially inactivated in cancer cells and might point to FOSL2 playing a regulatory role at these regions in the context of normal oesophageal cells.

Our results indicate that assessing AP1 and PEA3 transcription factor status might be a good indicator of OAC status. However, we could not detect any associations with disease stage or patient treatment regime. This suggests that the PEA3-AP1 regulatory module more likely contributes more generally to the cancer phenotype. In keeping with this observation, depletion of ETV1 and/or ETV4 causes an OAC cell growth defect ([16]; S1 Fig) and interfering with AP1 function also affects genes involved in growth and survival of OAC cells (Fig 3G). The PEA3-AP1 module and its regulatory network might therefore represent a target for therapeutic intervention. The PEA3-AP1 regulatory module had not been identified in these cancers before, most likely due to the complex nature of the AP1 and PEA3 family members involved in constituting this activity. Thus overexpression of any particular pairs of proteins would not necessarily have resulted in sufficient predictive power. This demonstrates the power of using ATAC-seq which reports on regulatory activities of transcription factor families rather than individual transcription factor activity. Moreover, the ability to apply ATAC-seq to low numbers of cells, makes this a particularly attractive method to use on the limited cell numbers available from patient-derived samples. The application of ATAC-seq to other cancers would therefore be a powerful approach to revealing new transcriptional regulatory events that contribute to tumourigenesis in these contexts. Moreover, ATAC-seq can be subjected to clustering analysis in a similar manner to RNA-seq to sub-partition cancers into different subtypes (Fig 6) and hence can be used in stratification strategies. By combining with single cell approaches [33], ATAC-seq has the potential to be able to uncover cancer heterogeneity and follow cancer evolution. This approach therefore has the potential to be used in both cancer diagnosis and stratification in addition to uncovering novel regulatory mechanisms that may prove amenable to therapeutic intervention.

## Materials and methods

### Ethics statement

Ethical approval for collection of oesophageal tissue samples from patients at the Royal Albert Edward Infirmary, Wigan and the Salford Royal Hospital were granted by the ethics committees at Wrightington, Wigan and Leigh NHS Foundation Trust (2007) and Salford Royal NHS Foundation Trust (2010) respectively (04/Q1410/57). Patient consent was obtained in written form and signed by the patient and doctor.

### Cell culture and tissue processing

The OE33 and OE19 cells were cultured in RPMI media supplemented with 10% foetal bovine serum. The FLO1, HET1A and 293T cells were cultured in DMEM culture media supplemented with 10% FBS. HEEPIC cells were grown on poly-l-lysine coated plates (2 μg/cm^2^) in epithelial cell media supplemented with 1% epithelial growth supplement. Biopsy tissue samples (~4 mm) were processed as described previously (Wiseman et al., 2015).

### RNA isolation, RT-qPCR and Fluidigm Biomark expression analysis

Total cellular RNA was isolated from cell line and clinical tissue samples as described previously [16]. When required, short interfering (si) RNAs directed against human *ETV1 or ETV4* (SMARTpools; Dharmacon), or a non-targeting pool (Dharmacon) were used in 24 hr transfections prior to RNA extraction as described previously [13]. For the validation experiments with either the SMARTpool or the single duplex siRNA construct against ETV1 (siGENOME human ETV1 siRNA, D-003801-04; Dharmacon), cells were left for 48 hrs after transfection. RT-qPCR was carried out as described previously [16] with the appropriate primer pairs (S8 Table). For analysis of the human tissue RNA samples, nanolitre volume RT-qPCR was performed using the Fluidigm Biomark HD system using EvaGreen chemistry according to the manufacturer’s instructions. The final relative expression was calculated by normalising to the geometric mean of the house-keeping genes *SDHA*, *ALAS1*, *GAPDH*, and *HMBS* using the delta CT method.

In knockdown experiments statistical significance was calculated using an unpaired two-tailed Student’s T test with a two sample equal variance. Gene expression data comparing expression in different groups of samples are represented with boxplots generated using Gene-e software. Outliers are not shown but represent values >1.5 interquartile ranges from the 25^th^ or the 75^th^ percentile (e.g 75^th^ percentile + (1.5 x IQR) and 25^th^ percentile–(1.5x IQR)). Statistical significance was assessed using a 2 tailed t-test calculated using StatPlus Microsoft Excel unless otherwise specified.

### Microarray and RNA-seq analysis

For expression microarray analysis of HET1A, HEEPIC, and OE33 cells (plus/minus siETV1/ETV4), biological triplicate RNA samples for each condition were processed. The raw intensity files (CEL) were generated by processing 500 ng of total RNA on Affymetrix HTA 2.0 arrays, according to the manufacturer's instructions (Affymetrix, Santa Clara, CA). The arrays were scanned through GENECHIP Scanner-7G (Affymetrix, CA). The CEL files generated by these arrays were converted into rma-gene-ful.chp and rma-alt-splice-dabg.chp files through Affymetrix Expression Console Software (version 1.3). The CHP files were analyzed through the Transcriptome Analysis Console v3.0 (TAC). Within the TAC software an excel spreadsheet for all conditions with the mean relative expression (n = 3) of all genes was generated. From this TAC also calculated fold change with between conditions. For all downstream analysis involving fold change genes this spreadsheet was used to generate gene lists for further analyses. Data are deposited in ArrayExpress (Accession number: E-MTAB-5163).

To generate samples for RNA-seq analysis, we transduced OE33 cells (3 biological replicates) with lentiviruses expressing a dominant-negative (DN) FOS (DN-FOS) construct (pInducer-DN-FOS) or lacking an insert (control)(pInducer) and grew for a further 12 hours. pInducer-DN-FOS was constructed by inserting a BamHI/EcoRI fragment from pBABE-puro-a-FOS/pAS2804 [21] into the same sites in pENTR1A (Addgene plasmid # 17398). The insert was then transferred to pInducer20 (a gift from Stephen Elledge; Addgene plasmid #44012; [34]) by Gateway cloning. Following transfection cells were treated with doxycycline (100 ng/ml) for 48 hours to induce DN-FOS expression and GFP positive cells were selected before harvesting RNA. The RNA-seq libraries are generated using the TruSeq stranded mRNA sample prep kit and 16 samples run on a NextSeq platform generating approximately 25 million reads per sample. Reads were first trimmed to remove Illumina adapter sequence using trimmomatic 0.3 [35]. Trimmed reads were aligned to the ensemble transcription (release 72) human genome 19 (hg19) using the RNA-Star aligner (version 2.3.0e) [36]. Differential expression analysis was carried out using Cuffdiff [37] in default settings. Data are deposited in ArrayExpress (Accession number: E-MTAB-5175).

### ChIP and ChIP-seq analysis

ChIP-qPCR and ChIP-seq were carried out as described previously [13]. For ChIP-qPCR for AP1, a JUN antibody (Abcam (ab31419)) was used and for histone acetylation a H3K27ac antibody (Abcam (ab4729)) was used. For ChIP-seq, 3x10^7^ cells, 3 μg antibody (ETV1; Abcam (ab81086)) and 30 μl Dynabeads were used per experiment. Parallel control experiments were run with ChIP-qPCR using rabbit IgG; Millipore (12–370). Library preparation was performed using the TruSeq ChIP Sample Preparation Protocol (Illumina) and DNA libraries were sequenced using the HiSeq 2500 (Illumina).

Sequencing tags/reads from the ETV1 ChIP-seq experiment in OE33 cells were aligned to the NBCI Build hg19 of the human genome with Bowtie v2.2.3 [38]. Up to two mismatches were allowed. Only reads with a mapping quality >q30 were retained. Peak calling was performed on individual replicates and merged datasets with MACS v2.1.0 software [39] using default parameters. Data are deposited in ArrayExpress (Accession number: E-MTAB-5168)

### ATAC-seq analysis

ATAC-seq data generation and analysis on OE19 and HEEPIC cell lines was performed as described previously for the HET1A, OE33 and FLO1 cell lines (Accession number E-MTAB-4209; [20]). To produce ATAC-seq libraries from human tissue samples fresh tissue was transported from the endoscopy department to the lab and processed within 1 hour of sampling. Alternatively, frozen tissue samples were used (surgical or endoscopic resections were immediately frozen down upon removal at -80°C, initially in liquid nitrogen, and then transferred to -80°C freezer). Frozen or fresh human tissue was first washed with 1x Dulbecco’s PBS. The tissue was then minced using scalpel and scissors. Minced tissue was lysed in 10 ml of fresh cold ATAC lysis buffer as described in [40] on ice. This suspension was briefly (5-10secs) vortexed every 10 mins during the lysis period and placed straight back onto ice. After 30 mins of lysis the lysed minced tissue was filtered first through 100 μm membrane then 20 μm membrane using low pressure vacuum driven filter units (Steriflipf filter units; Merck).The nuclei were then pelleted at 500g 4°C for 10 mins. The supernatant was removed and the pellet resuspended in 500 μl PBS and transferred to 1.5 ml microcentrifuge tube. The sample is again centrifuged at 500g 4°C for 10 mins to pellet the nuclei. The supernatant is removed and the nuclear pellet is resuspended in 25 μl nuclease free water before being quantified and then taken forward to the transposition reaction and processed as per the cell lines. Quantification of the nuclei is carried out by removing 2.5 μl of the resuspended nuclei and diluting to a total volume of 10 μl with 1x trypan blue and analysing on a haemocytometer.

Differentially accessible regions in cancer versus normal cells were identified by merging bam files of all of the conditions to be compared and recalling peaks using MACS2 [39]. To study higher confidence regions, a 500 bp window around the summit of the top 50,000 regions identified were then analysed for differential accessibility between different cell types using Cufflinks [41]. Differentially accessible regions were deemed to be those with a linear fold change of >5 fold and a p-value of <0.05 as determined by Cuffllinks. The differentially accessible regions were then taken forward for further analysis using a 500bp window centred on the summit of the region. Data are deposited in ArrayExpress (Accession number: E-MTAB-5169).

### Bioinformatics analysis

To visualise ATAC-seq data, normalised cleavage events across the differentially accessible regions were counted using HOMER [42] to produce heatmaps drawn using GENE-E (http://www.broadinstitute.org/cancer/software/GENE-E/). Hierachical clustering was carried out using this software, and all clustering was using one minus Pearson’s correlation unless otherwise specified. De novo motif discovery in ATAC-seq and ChIP-seq was carried out using HOMER [42] with the background normalised using–cpg parameter.

Gene annotation was performed using HOMER [42] to identify the closest gene to the ATAC-seq or ChIP-seq peak summits using the co-ordinates from the Refseq Hg19 v.37 protein coding list. The nearest gene was ascribed to the binding peak when the summit of the peak occurred within 100 kb upstream of the transcription start site (TSS). Gene ontology (GO) analysis was performed using the GREAT web application (http://bejerano.stanford.edu/great/public/html/) [43] using NBCI Build 37/hg 19 of the human genome. P-values for GO Terms shown are log_10_ of the binomial p-value generated using GREAT software [43].

Tag density heatmaps and profiles were generated using HOMER [42] using default settings and visualised using JavaTreeView 3.0 [44].

## Supporting information

S1 Fig Expression of PEA3 subfamily members and role in OAC cell growth. (PDF)

S2 Fig Validation of ETV1 ChIP-seq samples. (PDF)

S3 Fig Transcription factor motifs found at ETV1 binding regions. (PDF)

S4 Fig Validation of ETV1-mediated gene regulation. (PDF)

S5 Fig Reproducibility of ATAC-seq data. (PDF)

S6 Fig Functional categories of genes associated with differentially accessible chromatin regions. (PDF)

S7 Fig Transcription factor binding motifs associated with differentially accessible regions in cancer cells. (PDF)

S8 Fig Role of AP1 in determining H3K27 acetylation. (PDF)

S9 Fig Expression of putative directly regulated AP1 target genes in OAC tissue samples. (PDF)

S10 Fig Expression of PEA3 family and AP1 subunits in patient-derived samples. (PDF)

S11 Fig Integrative analysis of motif enrichment and transcription factor expression identifies likely regulatory transcription factors in normal oesophageal cells. (PDF)

S12 Fig Functional categories of genes associated with differentially accessible chromatin regions in OAC tissue samples. (PDF)

S13 Fig Expression of genes associated with differentially accessible chromatin regions in normal and OAC-derived cells. (PDF)

S14 Fig Footprinting at ETS and AP-1 motifs located in cancer cell-specific differentially accessible regions. (PDF)

S15 Fig Open chromatin levels in matched normal and tumour samples around ETV1 binding regions. (PDF)

S1 Table ETV1 binding regions identified from ChIP-seq analysis. Peaks are assigned to the gene with the nearest TSS.
(XLSX)

S2 Table Microarray analysis of gene expression in HET1A, HEEPIC and OE33 cells and OE33 cells following ETV1 and ETV4 depletion. Relative expression in each cell line/condition, and fold changes and associated P-values for the indicated comparisons are shown in tab 1. Tab 2 shows the genes that are significantly changed upon siETV1 treatment and the changes in their expression following treatment of OE33 cells with DN-FOS constructs.
(XLSX)

S3 Table Chromatin accessibility regions showing differential accessibility in OAC-derived cell lines. Columns J-Q show normalized Tn5 cutting frequency within each indicated 500 bp window in each of the indicated cell lines. Peaks are assigned to the gene with the nearest TSS.
(XLSX)

S4 Table RNAseq analysis of gene expression in OE33 cells following DN-FOS expression. Data are shown as averages of three experimental replicates.
(XLSX)

S5 Table Chromatin accessibility at ETV1 binding regions across different OAC-derived cell lines. Columns F-M show normalised Tn5 cutting frequency within each indicated ETV1 binding region in each of the indicated cell lines. The final column shows the clustering from Fig 4C.
(XLSX)

S6 Table Patient demographics. Information about samples used in ATAC-seq and for gene expression on the Fluidigm Biomark system are shown on separate tabs.
(XLSX)

S7 Table Chromatin accessibility regions showing differential accessibility in OAC-derived patient samples. Columns H-P show normalised Tn5 cutting frequency within each indicated chromatin regions in each of the indicated tissue samples from normal tissue (N) or OAC tumour samples (T). Regions are included that show significant differential accessibility (5 fold difference in tag counts; p-value <0.05) between the normal and the cancer samples.
(XLSX)

S8 Table RT-qPCR and ChIP-qPCR primer pairs. (XLSX)
