# Supplementary material for: Open chromatin profiling identifies AP1 as a transcriptional regulator in oesophageal adenocarcinoma
Source: PLoS Genet. 2017 Aug 31;13(8):e1006879. doi: 10.1371/journal.pgen.1006879 (PMC5578490; doi:10.1371/journal.pgen.1006879)
Supplement: S1 Fig — (PDF) [file pgen.1006879.s001.pdf]

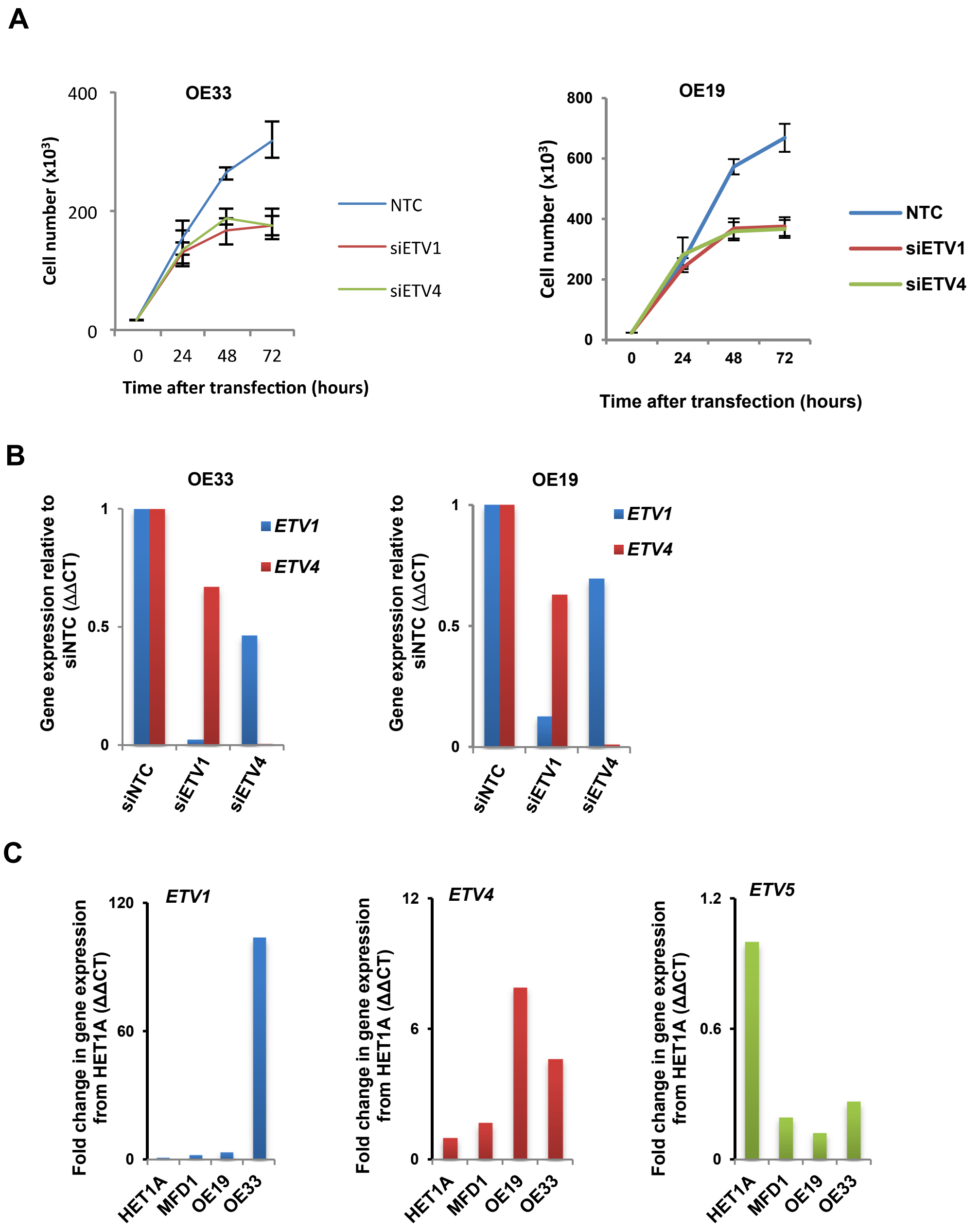

S1 Fig. Expression of PEA3 subfamily members and role in OAC cell growth. (A) Growth curves of OE33 (left) and OE19 (right) cells following treatment with a non-targeting control (MTC) siRNA or siRNAs targeting either *ETV1* or *ETV4* (n=3). (B) RT-qPCR validation of the efficiency of siRNA-mediated *ETV1* and *ETV4* depletion. (C) RT-qPCR analysis of *ETV1*, *ETV4* and *ETV5* expression in the indicated cell lines. Data are shown relative to the expression in HET1A cells (taken as 1).
