# Supplementary material for: Open chromatin profiling identifies AP1 as a transcriptional regulator in oesophageal adenocarcinoma
Source: PLoS Genet. 2017 Aug 31;13(8):e1006879. doi: 10.1371/journal.pgen.1006879 (PMC5578490; doi:10.1371/journal.pgen.1006879)
Supplement: S2 Fig — (PDF) [file pgen.1006879.s002.pdf]

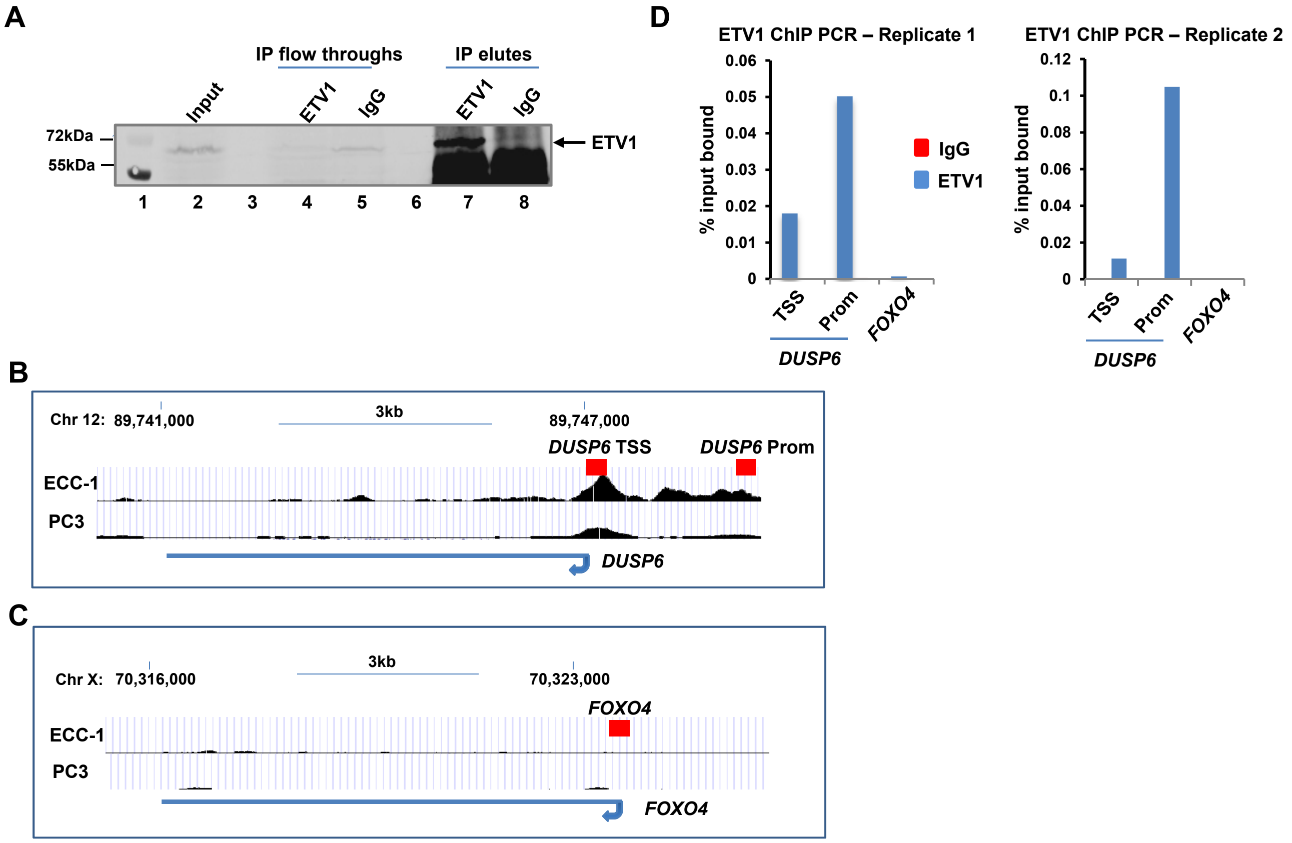

S2 Fig. Validation of ETV1 ChIP-seq samples (A) Western blot of ETV1 levels in immunoprecipitation (IP) with ETV1 antibody or control non-specific IgG performed under ChIP conditions. Input (3.3%; lane 2), flow through (FT) after antibody binding (3.3%; lanes 4 and 5) and eluates from the IP (100%; lanes 7 and 8) are shown. ETV1 is specifically depleted from the lysate and retained on the antibody-conjugated beads with the ETV1 antibody. Lanes 3 and 6 are empty. (B and C) UCSC genome browser tracks of the genomic regions surrounding positive (*DUSP6*) and negative (*FOXO4*) binding regions identified using two PEA3 family ChIP-seq datasets from endometrial adenocarcinoma for ETV4 (ECC-1; Gertz et al., 2013) or prostate cancer for ETV1 (PC3; [17, 18]) cells. (D) ChIP-qPCR of the positive and negative control regions for both ETV1 IP (blue) and control IgG IP (red) used for the two ETV1 ChIP-seq experiments. The targets for the ChIP PCR primers are shown in (B) and (C) by red bars. Data are shown relative to input chromatin levels.
