# Supplementary material for: Open chromatin profiling identifies AP1 as a transcriptional regulator in oesophageal adenocarcinoma
Source: PLoS Genet. 2017 Aug 31;13(8):e1006879. doi: 10.1371/journal.pgen.1006879 (PMC5578490; doi:10.1371/journal.pgen.1006879)
Supplement: S3 Fig — (PDF) [file pgen.1006879.s003.pdf]

ETV1 Target Regions (n=498)

| | Motif | % targets | % background | p value |
|---|---|---|---|---|
| AP-1 | 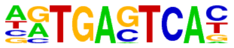 | 65.06 | 11.25 | $1x10^{-177}$ |
| ETS | 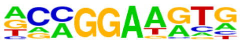 | 75.70 | 19.42 | $1x10^{-161}$ |
| TCF7L2 | 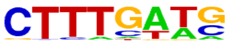 | 28.50 | 13.81 | $1x10^{-16}$ |
| TEAD2 | 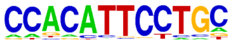 | 8.84 | 2.09 | $1x10^{-14}$ |
| RUNX | 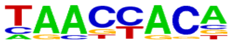 | 18.27 | 7.77 | $1x10^{-13}$ |

**S3 Fig. Transcription factor motifs found at ETV1 binding regions.** Top five most significant motifs from *de novo* motif discovery at the ETV1 binding regions identified by ChIP-seq.
