# Supplementary material for: Open chromatin profiling identifies AP1 as a transcriptional regulator in oesophageal adenocarcinoma
Source: PLoS Genet. 2017 Aug 31;13(8):e1006879. doi: 10.1371/journal.pgen.1006879 (PMC5578490; doi:10.1371/journal.pgen.1006879)
Supplement: S4 Fig — (PDF) [file pgen.1006879.s004.pdf]

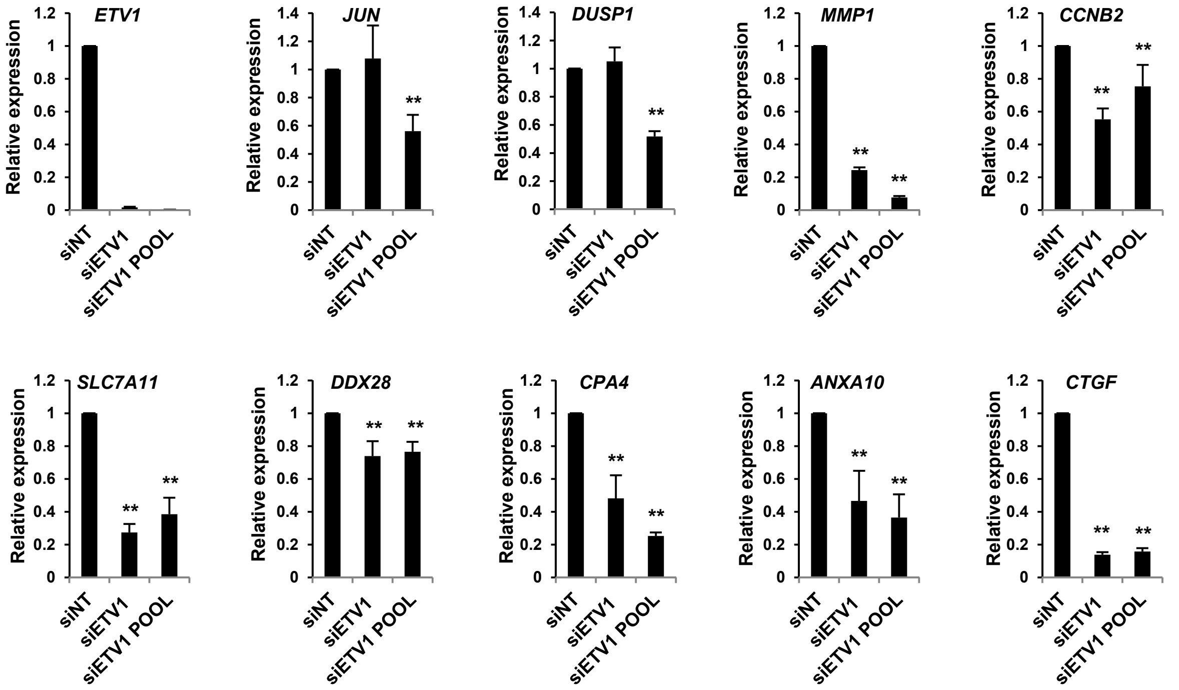

S4 Fig. Validation of ETV1-mediated gene regulation. RT-qPCR analysis of the indicated genes in OE33 cells transfected with either a non-targeting (NT) control siRNA pool, a pool of siRNA duplexes targeting ETV1 or a single siRNA duplex which is not found in this pool. Data were normalised to *RPLP0* expression and are shown as means of independent experiments (n=3) relative to the control cells (taken as "1"). Error bars represent standard deviations. **= P-value <0.01 (t-test). With the exception of *JUN* and *DUSP1* all of the genes respond in a similar manner to the pool of siRNAs and the new unique single siRNA against ETV1.
