# Supplementary material for: Open chromatin profiling identifies AP1 as a transcriptional regulator in oesophageal adenocarcinoma
Source: PLoS Genet. 2017 Aug 31;13(8):e1006879. doi: 10.1371/journal.pgen.1006879 (PMC5578490; doi:10.1371/journal.pgen.1006879)
Supplement: S5 Fig — (PDF) [file pgen.1006879.s005.pdf]

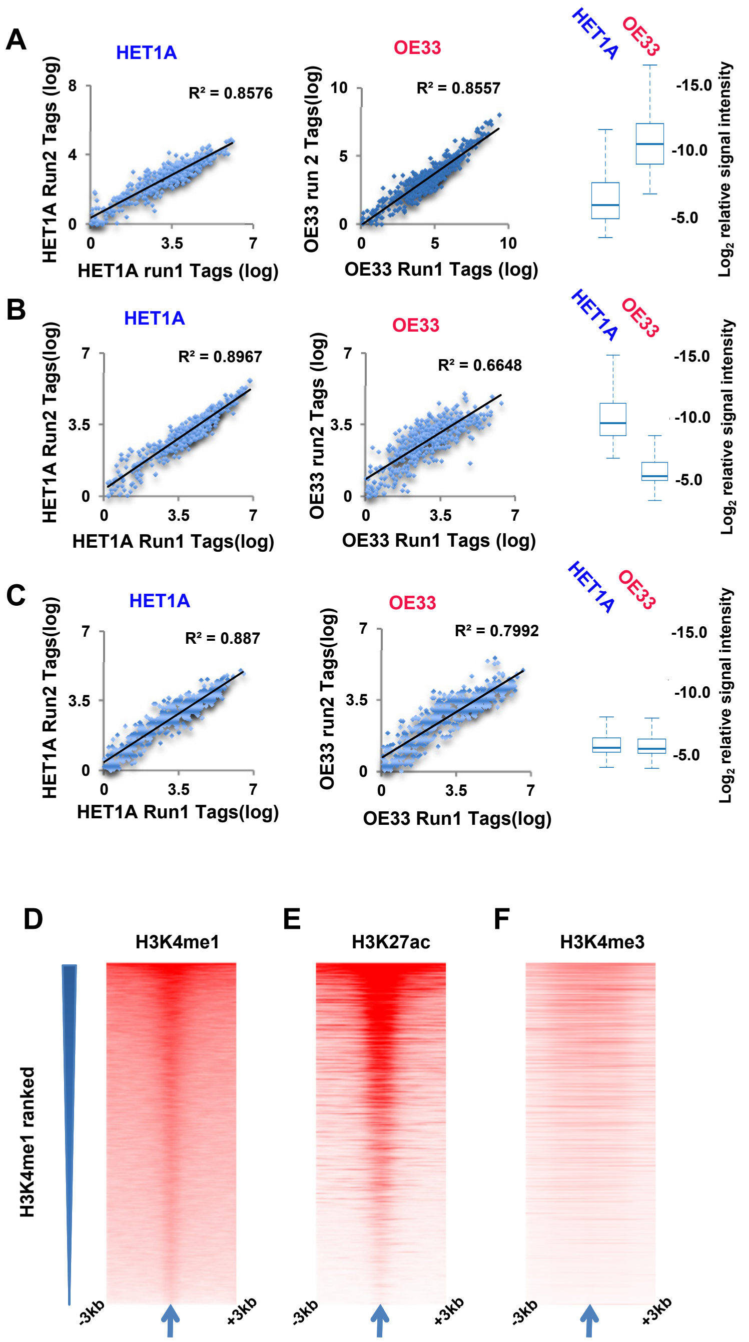

S5 Fig. Reproducibility of ATAC-seq data (A-C) Correlation plots of the normalised ATAC-seq tag count in replicate experiments at the proximal promoters of the top 500 genes in different categories which are differentially expressed between OE33 and HET1A cells. Data are shown for genes which differ in each direction and are (A) higher in cancer (OE33) or (B) higher in normal (HET1A). 500 genes which show no difference in expression between cell lines are also shown (C). The expression of each category of genes in the two cell lines are shown as boxplots on the right and represent the relative signal intensity ($\log_2$) from microarray experiments. Horizontal lines represent the median expression values. (D-F) Heatmaps of tag density profiles of histone modifications found in cells from normal oesohageal epithelial tissue are shown in a 6 kb window around the summit (blue arrow) of distal intergenic ATAC-seq peaks from merged normal cell line analysis (n=18,372). Peaks in all heatmaps are ranked according to the density of the H3K4me1 ChIP signal. GEO accession numbers (GSM1120349 H3k4me1, GSM1227069 H3K4me3, GSM1013127 H3K27ac). A large proportion of distal intergenic peaks are enriched for H3K4me1, and H3K27ac which is consistent with them representing areas of active chromatin. H3K4me3 levels are relatively low across these regions, consistent with their annotation as promoter distal regions.
