# Supplementary material for: Open chromatin profiling identifies AP1 as a transcriptional regulator in oesophageal adenocarcinoma
Source: PLoS Genet. 2017 Aug 31;13(8):e1006879. doi: 10.1371/journal.pgen.1006879 (PMC5578490; doi:10.1371/journal.pgen.1006879)
Supplement: S6 Fig — (PDF) [file pgen.1006879.s006.pdf]

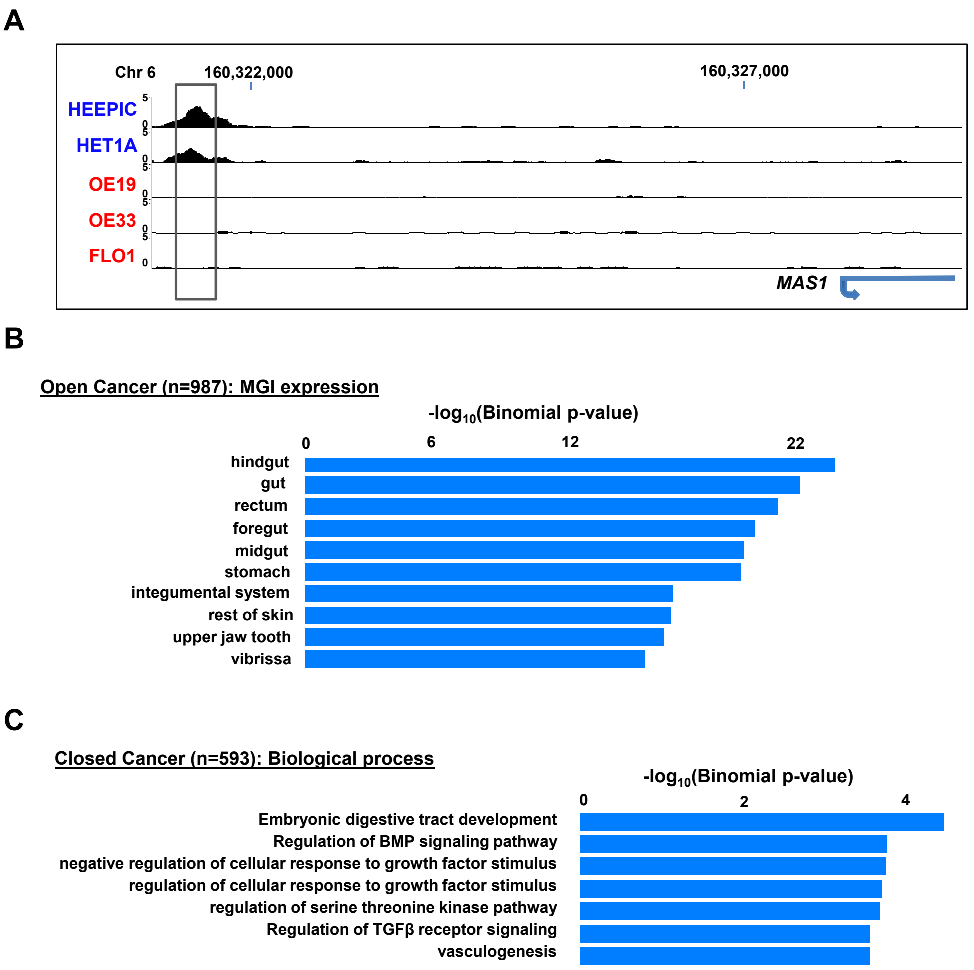

S6 Fig. Functional categories of genes associated with differentially accessible chromatin regions. (A) UCSC browser track showing open chromatin regions at the *MAS1* locus in the indicated cell lines. An intragenic (grey box) peak is highlighted. (B and C) The top gene ontology terms of the genes associated with the differentially accessible regions more open in cancer cells (B; MGI expression category) or more open in normal cells (C; Biological process category).
