# Supplementary material for: Open chromatin profiling identifies AP1 as a transcriptional regulator in oesophageal adenocarcinoma
Source: PLoS Genet. 2017 Aug 31;13(8):e1006879. doi: 10.1371/journal.pgen.1006879 (PMC5578490; doi:10.1371/journal.pgen.1006879)
Supplement: S7 Fig — (PDF) [file pgen.1006879.s007.pdf]

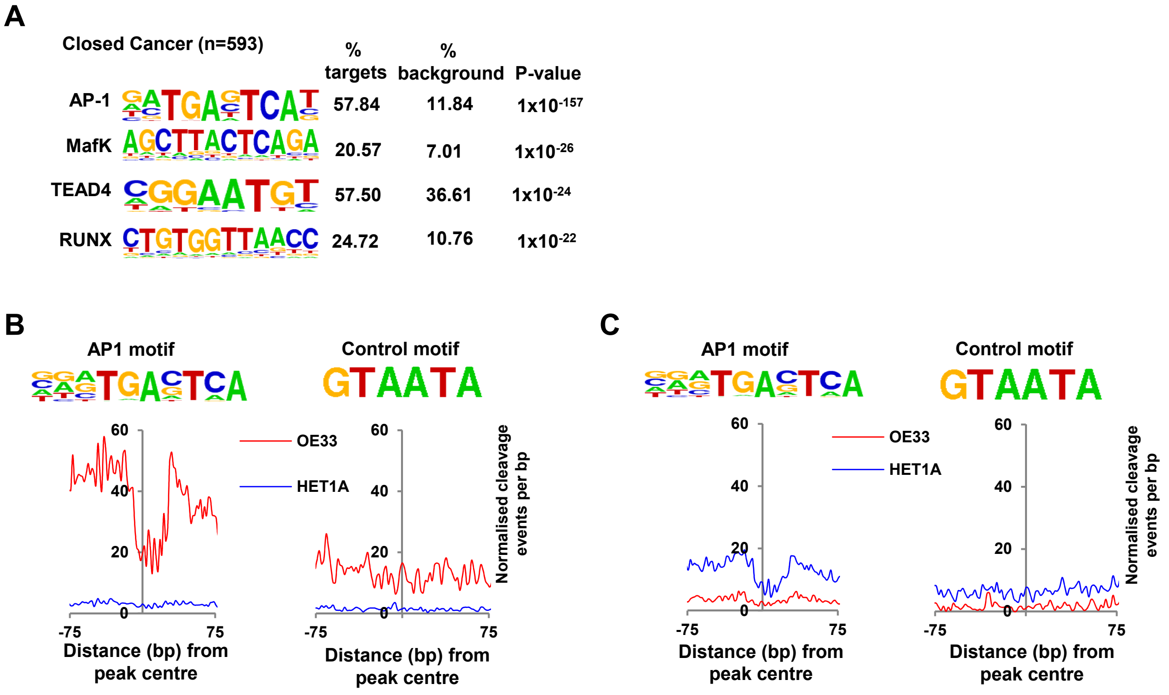

S7 Fig. Transcription factor binding motifs associated with differentially accessible regions in cancer cells. (A) Top four motifs from *de novo* motif discovery at the 593 differentially accessible chromatin regions (+/- 250 bp from the centre) which are more open in normal cells. (B and C) Plots of normalised Tn5 cleavage events +/-75bp from the motif centre (blue arrow) around the AP-1 motifs found in differentially accessible chromatin regions that are more open in cancer cells (B) or more open in normal cells (C). The same data centered on a control hexameric GTAATA motif if shown on the right and shows no evidence of a transcription factor footprint. Data are plotted from OE33 (red) and HET1A (blue) cells.
