# Supplementary material for: Open chromatin profiling identifies AP1 as a transcriptional regulator in oesophageal adenocarcinoma
Source: PLoS Genet. 2017 Aug 31;13(8):e1006879. doi: 10.1371/journal.pgen.1006879 (PMC5578490; doi:10.1371/journal.pgen.1006879)
Supplement: S8 Fig — (PDF) [file pgen.1006879.s008.pdf]

A

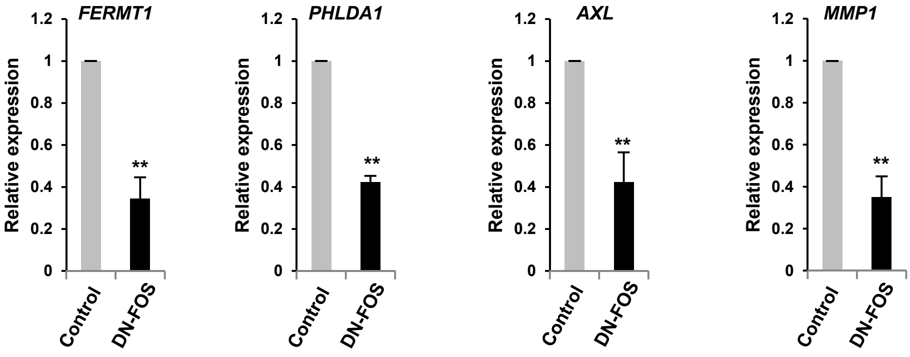

B

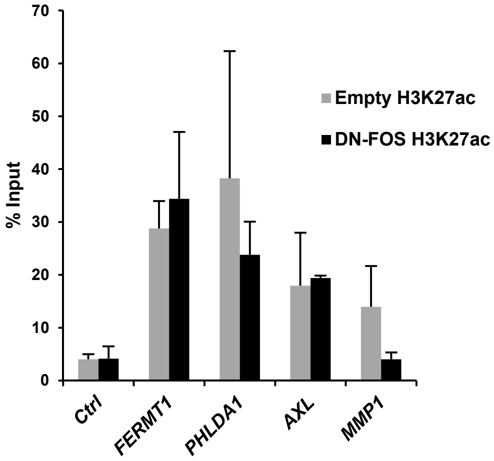

S8 Fig. Role of AP1 in determining H3K27 acetylation. (A) RT-qPCR analysis of the indicated genes in OE33 cells transfected with empty lentivirus (control) or a lentivirus encoding DN-FOS. Data were normalised to *RPLP0* expression and are shown as means of independent experiments (n=3) relative to the control cells (taken as “1”). Error bars represent standard deviations. **= P-value <0.01 (t-test). (B) ChIP-qPCR analysis of H3K27 acetylation (ac) levels in accessible chromatin regions associated with the indicated genes in OE33 cells. Cells were treated in parallel to the experiments in (A) with empty lentivirus (control) or a lentivirus encoding DN-FOS. Non-specific IgG is used as a control (ctrl) and intron 1 from the *ABHD12* locus is a control region not bound by AP1. Data are shown as means ±SD (n=3).
