# Supplementary material for: Open chromatin profiling identifies AP1 as a transcriptional regulator in oesophageal adenocarcinoma
Source: PLoS Genet. 2017 Aug 31;13(8):e1006879. doi: 10.1371/journal.pgen.1006879 (PMC5578490; doi:10.1371/journal.pgen.1006879)
Supplement: S9 Fig — (PDF) [file pgen.1006879.s009.pdf]

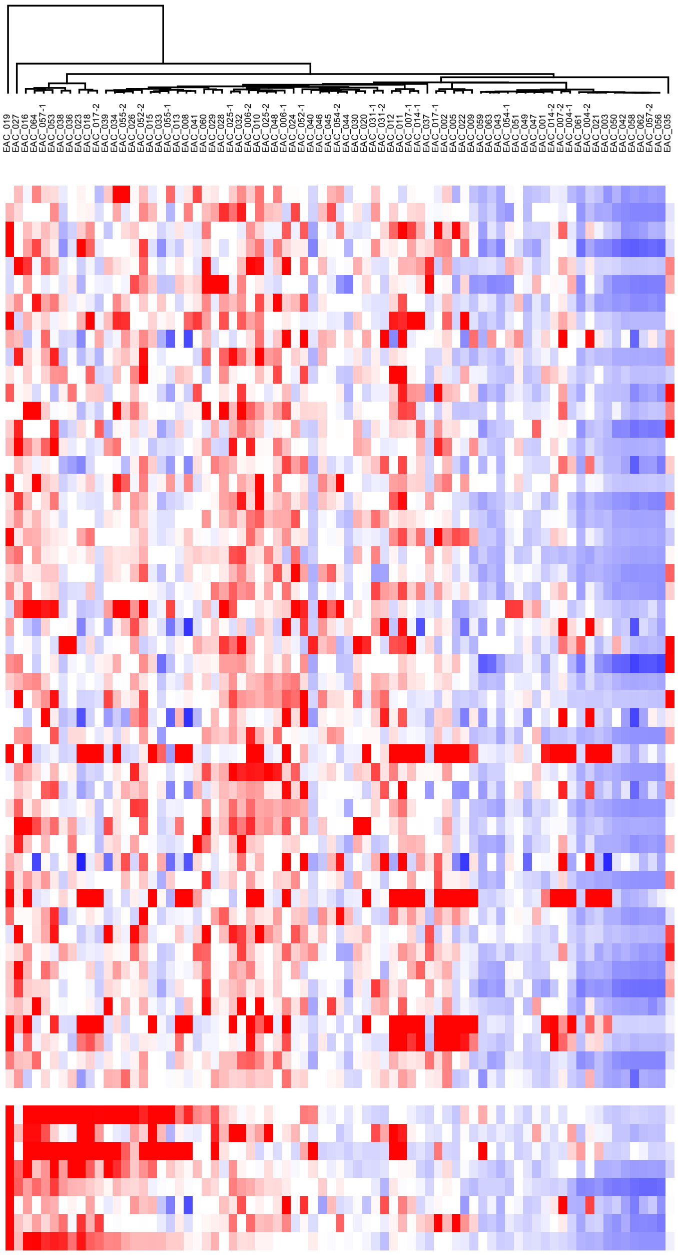

Gene Name	Fold change in expression; DN-FOS to Normal (log_2)
CCSER2	0.3834
PFKP	0.4091
C1orf116	0.4318
PMVK	0.5123
SEMA3C	0.5137
FXYD5	0.523
PRKAG2	0.5336
CAV2	0.5559
GTF2IRD1	0.5565
MET	0.5686
KYNU	0.5783
ARL4C	0.6678
SEMA3A	0.6808
MYO18A	0.7618
ARL4A	0.7681
ITGA6	0.789
SDCBP2	0.7901
GPATCH1	0.7932
MRPL15	0.8093
OSTF1	0.8258
HNRNPD	0.8392
ECH1	0.8737
INPP4B	0.9783
GATA6	0.9885
IFRD1	1.01
IGFBP3	1.06
GLTSCR1	1.19
NSMCE2	1.24
ACSL5	1.31
LAMA3	1.32
NPAS2	1.35
KLK5	1.37
ZFP36L2	1.45
DOCK4	1.46
DHX36	1.47
CLDN12	1.5
MAP4K4	1.51
SMURF1	1.57
OXR1	1.6
DSC3	1.61
FAM102A	1.8
GOLIM4	1.85
PHTF2	2.04
LAPTM4A	2.18
URI1	2.22
SLMO2	2.25
KRT15	2.36
DAPP1	3.03
MDH2	3.33
LBR	4.08
FOS	NaN
FOSL1	NaN
FOSB	NaN
JUN	NaN
JUND	NaN
JUNB	NaN
FOSL2	NaN
AP1 av	NaN

S9 Fig. Expression of putative directly regulated AP1 target genes in OAC tissue samples. Expression of putative AP1 target genes across OAC-derived tissue samples (microarray data, GSE13898; Kim et al., 2010). AP1 target genes are selected based on their association with nearby regions that are both more open in cancer cells and also contain an AP1 motif and in addition, exhibit reduced expression following DN-FOS expression (>1.3 fold). Data are row Z normalised and subjected to Eucladian clustering and three main subclusters are highlighted (SC1-3). SC3 delineates samples that show low level AP1 expression and low level target gene expression. Data are the same as in Fig. 3H, except that gene names and fold changes in expression due to DN-FOS expression are included on the right.
