# Supplementary material for: Open chromatin profiling identifies AP1 as a transcriptional regulator in oesophageal adenocarcinoma
Source: PLoS Genet. 2017 Aug 31;13(8):e1006879. doi: 10.1371/journal.pgen.1006879 (PMC5578490; doi:10.1371/journal.pgen.1006879)
Supplement: S10 Fig — (PDF) [file pgen.1006879.s010.pdf]

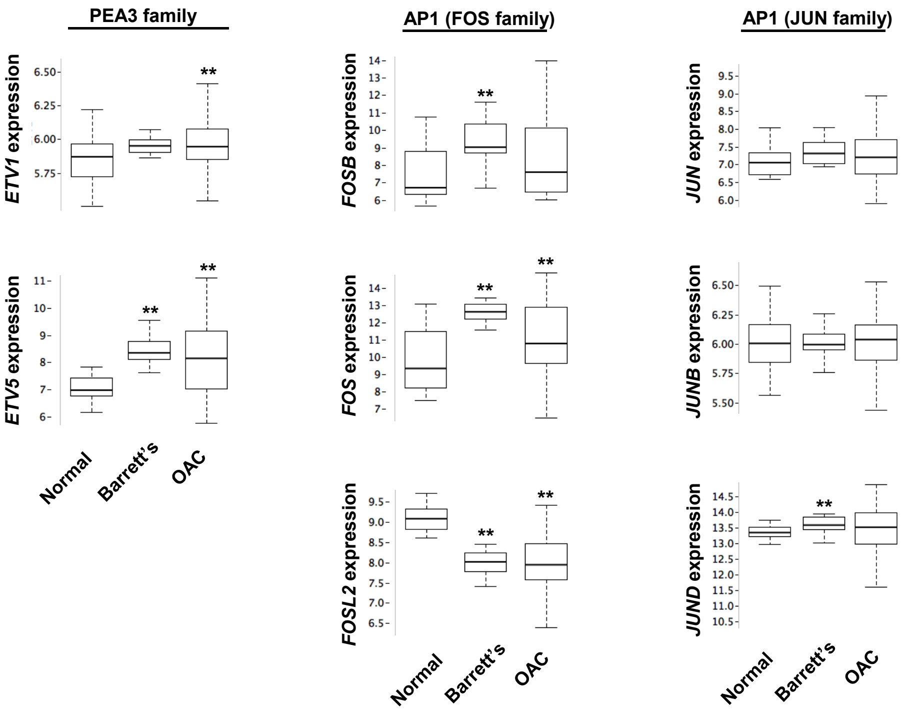

S10 Fig. Expression of PEA3 family and AP1 subunits in patient-derived samples. Box plots of expression (log2) of the indicated genes from microarray data [4] from normal, Barrett's or OAC-derived samples. Horizontal line indicates median values. ** P-value <0.05 (t test).
