# Supplementary material for: Open chromatin profiling identifies AP1 as a transcriptional regulator in oesophageal adenocarcinoma
Source: PLoS Genet. 2017 Aug 31;13(8):e1006879. doi: 10.1371/journal.pgen.1006879 (PMC5578490; doi:10.1371/journal.pgen.1006879)
Supplement: S11 Fig — (PDF) [file pgen.1006879.s011.pdf]

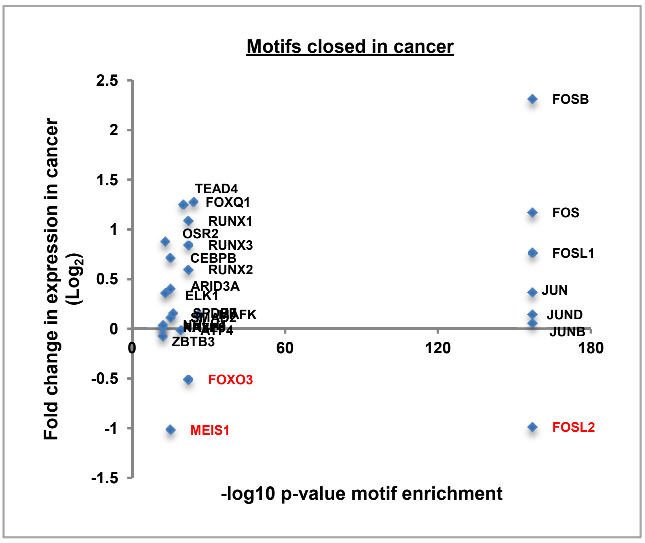

S11 Fig. Integrative analysis of motif enrichment and transcription factor expression identifies likely regulatory transcription factors in normal oesophageal cells. To determine putative regulatory TFs, the enrichment of transcription factor motifs (-$\log_{10}$ binomial P-value)(x-axis) is plotted against fold difference ($\log_2$) in the mean gene expression (from microarray data; [4]) of the transcription factors which potentially bind to the motif, in normal cells compared to cancer cells (y-axis). Those highlighted in red show are deemed significant (linear 1.5 fold change in the same direction as change in accessibility of the associated motif).
