# Supplementary material for: Open chromatin profiling identifies AP1 as a transcriptional regulator in oesophageal adenocarcinoma
Source: PLoS Genet. 2017 Aug 31;13(8):e1006879. doi: 10.1371/journal.pgen.1006879 (PMC5578490; doi:10.1371/journal.pgen.1006879)
Supplement: S12 Fig — (PDF) [file pgen.1006879.s012.pdf]

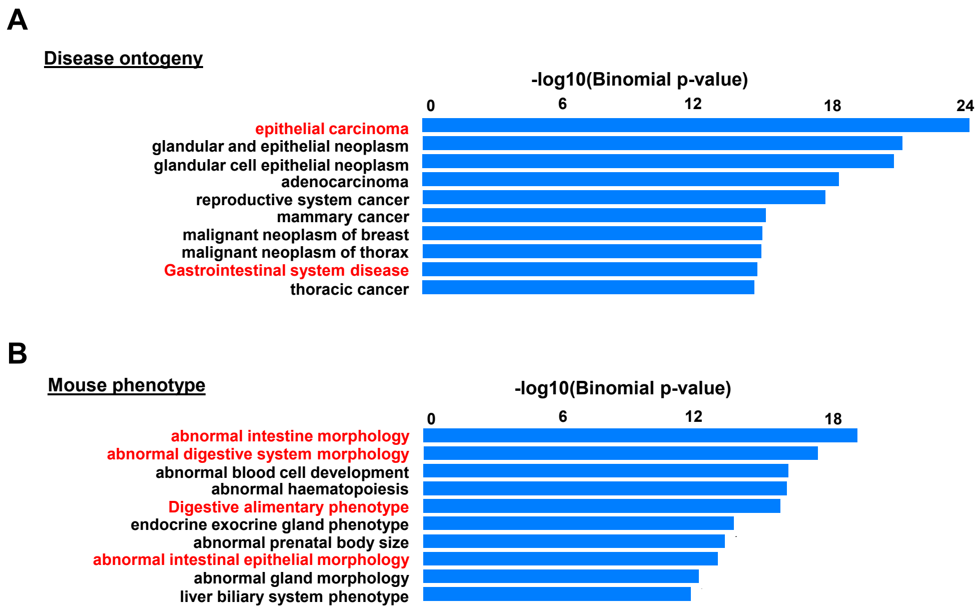

S12 Fig. Functional categories of genes associated with differentially accessible chromatin regions in OAC tissue samples. The top 10 GO terms from the (A) disease ontology and (B) mouse phenotype categories determined for the genes with the nearest TSS and within 100 kb of the differentially accessible ATAC-seq peaks identified from human tissue samples. Features particularly relevant to the clinical phenotype are highlighted in red font.
