# Supplementary material for: Open chromatin profiling identifies AP1 as a transcriptional regulator in oesophageal adenocarcinoma
Source: PLoS Genet. 2017 Aug 31;13(8):e1006879. doi: 10.1371/journal.pgen.1006879 (PMC5578490; doi:10.1371/journal.pgen.1006879)
Supplement: S13 Fig — (PDF) [file pgen.1006879.s013.pdf]

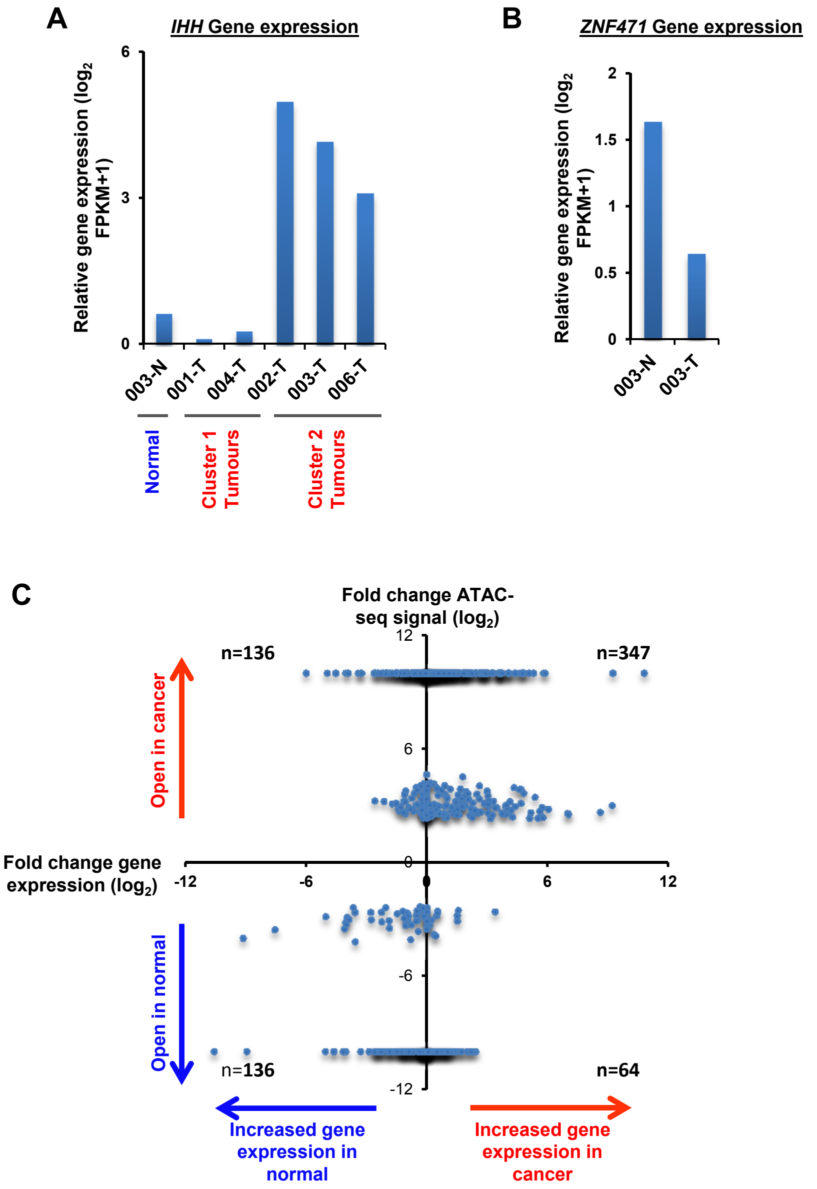

S13 Fig. Expression of genes associated with differentially accessible chromatin regions in normal and OAC-derived cells. (A) The relative expression (FPKM) of *IHH* in the indicated tissue samples from RNA-seq data. (B) The relative expression (FPKM) of *ZNF471* for the matched paired normal sample sequenced. The increased *IHH* expression in tumour cells is consistent with the appearance of open chromatin regulatory regions in the same cells. Reciprocally, the reduced expression of *ZNF471* matches the reduced accessibility of its regulatory regions in tumour cells. (C) Scatter plot showing a comparison between the changes in open chromatin levels from the ATAC-seq signal and gene expression levels of the associated genes from RNA-seq. Data are shown for the patient sample 003-T and are shown as log_2 fold change between the tumour (T) and normal (N) samples. Only regions which show a 5 fold linear change in accessibility between tumour and normal samples were used. Genes associated with two or more peaks that show reciprocal increases and decreases in accessibility were discarded leaving 569 genes.
