# Supplementary material for: Open chromatin profiling identifies AP1 as a transcriptional regulator in oesophageal adenocarcinoma
Source: PLoS Genet. 2017 Aug 31;13(8):e1006879. doi: 10.1371/journal.pgen.1006879 (PMC5578490; doi:10.1371/journal.pgen.1006879)
Supplement: S14 Fig — (PDF) [file pgen.1006879.s014.pdf]

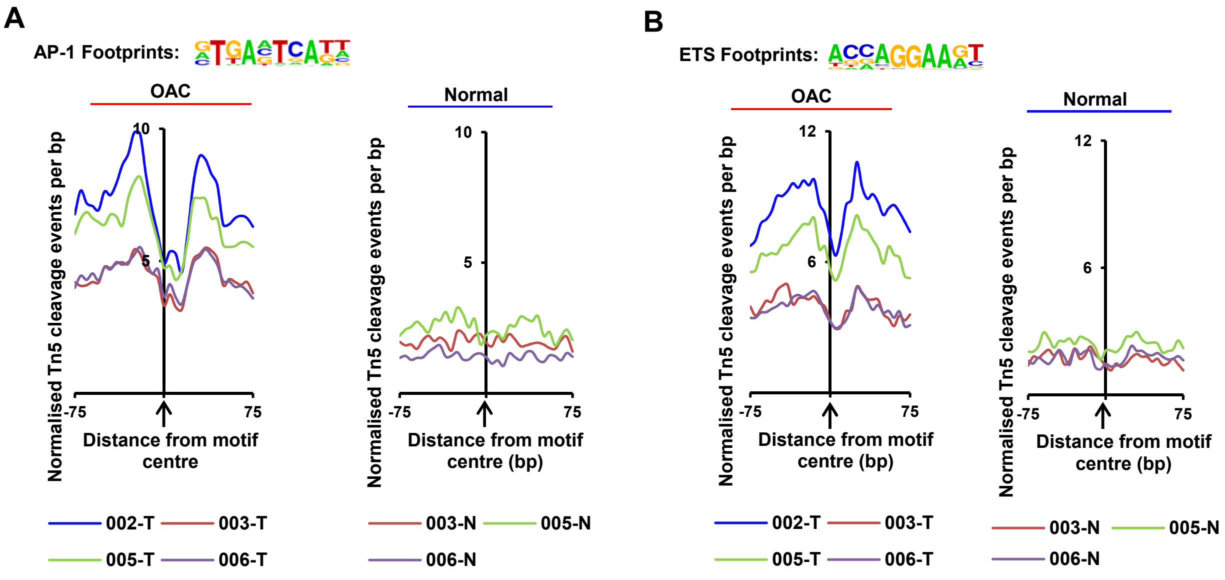

S14 Fig. Footprinting at ETS and AP-1 motifs located in cancer cell-specific differentially accessible regions. (A and B) Plots of normalised Tn5 cleavage events +/-75 bp from the AP-1 (A) or ETS (B) motif centres (arrows). The left plots are for cluster 2 tumour samples and the right plots are from all normal samples. There are clearly better defined footprints in the cancer-cell derived ATAC-seq data.
