# Supplementary material for: Open chromatin profiling identifies AP1 as a transcriptional regulator in oesophageal adenocarcinoma
Source: PLoS Genet. 2017 Aug 31;13(8):e1006879. doi: 10.1371/journal.pgen.1006879 (PMC5578490; doi:10.1371/journal.pgen.1006879)
Supplement: S15 Fig — (PDF) [file pgen.1006879.s015.pdf]

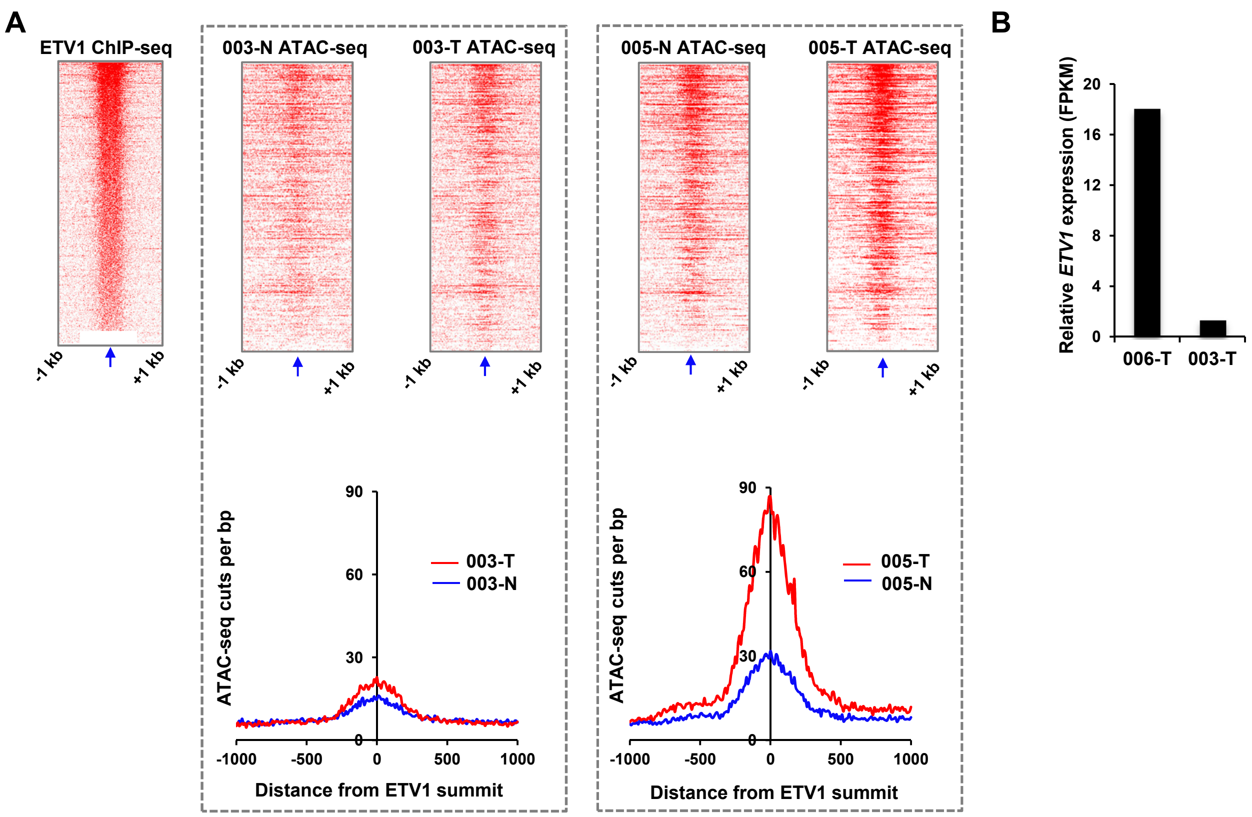

S15 Fig. Open chromatin levels in matched normal and tumour samples around ETV1 binding regions. (A) Heatmaps (top) and average ATAC-seq cleavage events (bottom) of ATAC-seq tag density in normal (blue line) and tumour (red line) samples from patient 003 and 005. Data are shown in a +/- 1 kb region relative to the summit of the ETV1 binding peaks defined by ChIP-seq in OE33 cells. Regions in the heatmaps are ranked according to ETV1 ChIP-seq signal (shown on the left). (B) Relative expression of *ETV1* in RNA-seq data (RPKM) from patients 003 and 006. The reduced enrichment of open chromatin observed around ETV1 binding sites in the cancer sample from patient 003 is consistent with the much reduced expression of *ETV1* in the cancers derived from this patient.
